# Synergistic anti-*Helicobacter pylori* efficacy of a molecularly identified *Limosilactobacillus fermentum* isolate in combination with multiple antibiotics

**DOI:** 10.1186/s12866-026-05350-8

**Published:** 2026-07-04

**Authors:** Menna M. M. Mohammed Ali, Hala Mohamed Abu Shady, Sayed. M. M, Hayam A. E. Sayed

**Affiliations:** 1 Department of Microbiology, Faculty of Science, Ain Shams University, Cairo, 11566 Egypt; 2 Department of internal medicine and gastroenterology, Faculty of medicine, Ain Shams University, Cairo, 11566 Egypt

**Keywords:** Helicobacter pylori, Limosilactobacillus fermentum, Antibiotic synergy, Bacteriocins, like compounds

## Abstract

**Background:**

*Helicobacter pylori* (*H. pylori*) is a Gram-negative gastric pathogen resistant to the acidic stomach environment through urease-mediated neutralization, enabling colonization of the gastric mucosa. It is a major cause of gastritis, peptic ulcer disease, and gastric cancer, and was classified as a Class I carcinogen by the International Agency for Research on Cancer (IARC) in 1994. Rising antibiotic resistance has limited the efficacy of conventional therapies, highlighting the need for alternative or adjunct antimicrobial strategies.

**Methods:**

Forty lactic acid bacteria (LAB) isolates were screened for anti-*H. pylori* activity against twenty clinical *H. pylori* isolates previously recovered from gastric biopsies of Egyptian patients using neutralized cell-free supernatants (CFSs). The CFSs were evaluated alone and in combination with standard therapeutic antibiotics. The most potent CFS was subsequently evaluated for cytotoxic activity against colorectal adenocarcinoma (Caco-2) cells as a preliminary assessment of its potential anticancer-related properties. The CFS was then subjected to fast protein liquid chromatography (FPLC), and the active fraction was further analyzed by sodium dodecyl sulfate–polyacrylamide gel electrophoresis (SDS-PAGE) to estimate its molecular weight. Following verification of its probiotic characteristics, the producer strain was identified by 16 S rRNA gene sequencing.

**Results:**

Nine out of forty LAB isolates demonstrated anti-*H. pylori* activity, with minimum inhibitory concentration (MIC) and minimum bactericidal concentration (MBC) values ranging from 93.7 to 750 µg/mL. Combination assays demonstrated isolate-dependent interactions, with synergistic effects observed with clarithromycin (CLR), amoxicillin (AX), metronidazole (MET), tetracycline (TE), rifampicin (RA), and levofloxacin (LEV) against certain *H. pylori* isolate groups. *L. fermentum* strain M98, identified as the most potent isolate, exhibited the highest inhibitory activity both alone and in combination with antibiotics. The protein-enriched precipitate containing a putative bacteriocin exhibited cytotoxic activity against Caco-2 cells, with an IC₅₀ value of 19.73 ± 0.16 µg/mL. FPLC of its CFS yielded 22 protein fractions, three of which exhibited anti-H. pylori activity, suggesting the presence of bacteriocin-like compounds. Subsequent SDS-PAGE analysis of the active fraction revealed a prominent protein band of approximately 34 kDa.

**Conclusion:**

The CFS of *L. fermentum* strain M98 exhibits significant in vitro anti-*H. pylori* activity and enhances the efficacy of tested antibiotics, supporting its potential as an adjunct therapeutic agent against antibiotic-resistant *H. pylori*. In addition, LAB-precipitated proteins containing a putative bacteriocin demonstrated cytotoxic activity against Caco-2 cells, suggesting the presence of bioactive compounds with potential therapeutic value. Further in vivo investigations are warranted to confirm their safety, efficacy, and potential biomedical applications.

**Supplementary Information:**

The online version contains supplementary material available at 10.1186/s12866-026-05350-8.

## Background

*Helicobacter pylori* (*H. pylori*) is a Gram-negative pathogen capable of surviving in the acidic environment of the stomach by producing urease, which neutralizes gastric acid and facilitates colonization of the gastric mucous layer. This adaptation contributes to its persistence and pathogenicity in the human host. In 1994, *H. pylori* was classified as a Group 1 carcinogen by the International Agency for Research on Cancer (IARC), confirming its causal role in gastric malignancies [1]. *H. pylori* is commonly acquired during childhood, and persistent infection may lead to gastric adenocarcinoma and mucosa-associated lymphoid tissue (MALT) lymphoma in adulthood. It is recognized as one of the most important infectious causes of cancer worldwide, with approximately 75–80% of non-cardia gastric cancers attributable to *H. pylori* infection. The global prevalence of *H. pylori* infection is estimated at approximately 40–50%, with marked geographic variation ranging from 20 to 30% in high-income countries to over 70% in low-income regions. Infection is often asymptomatic, particularly in children, making accurate prevalence estimation challenging. Reported estimates suggest a childhood prevalence of approximately 30–35%, while adult prevalence ranges from approximately 24% to over 70%, with a pooled global prevalence close to 50% [2–3]. In Egypt, prevalence remains high, with infection rates of approximately 64.6% in children and 52% in adults, highlighting a substantial public health burden in the region [4–5]. Although eradication therapy is generally effective, widespread antibiotic use has contributed to the emergence of antibiotic-resistant *H. pylori* strains. Increasing resistance has been associated with treatment failure and recurrence in multiple studies [2, 6]. Therefore, there is a growing need for improved therapeutic strategies and alternative or adjunct approaches to enhance eradication success.

Probiotics “live microorganisms that confer health benefits to the host when administered in adequate amounts have been widely studied as a therapeutic agent against different pathogens, including *H. pylori* and other gastrointestinal pathogens [7]. They represent an excellent choice, as it was reported in many recent reviews and umbrella analyses that probiotics exhibit a therapeutic effect against *H. pylori* as well as act effectively as an antibiotic adjunct, improving the eradication rate and reducing the treatment-related side effects [8–10].

Probiotics exert their beneficial effects through multiple mechanisms, including the production of bioactive metabolites such as organic acids, short-chain fatty acids, hydrogen peroxide, and bacteriocins [11]. Among these, bacteriocins are particularly important due to their targeted antimicrobial activity, which enables inhibition of specific pathogens including *Helicobacter pylori*,* Clostridioides difficile*,* Vibrio parahaemolyticus*,* Escherichia coli* O157, and *Staphylococcus aureus* [12–13].

Bacteriocins and bacteriocin-like compounds produced by lactic acid bacteria have also attracted increasing interest for their potential anticancer-related properties. Several studies have reported that these compounds may exert cytotoxic effects on cancer cell lines through mechanisms such as disruption of membrane integrity, induction of apoptosis, and modulation of cell cycle progression. Although initially considered to have a narrow antimicrobial spectrum, recent evidence suggests that bacteriocins may exhibit broader biological activities, including effects on eukaryotic cells under in vitro conditions [14].

Therefore, this study aimed to evaluate the anti-*H. pylori* activity of locally isolated LAB strains, investigate their potential synergistic interactions with antibiotics used in *H. pylori* eradication therapy, and characterize the cell-free supernatant (CFS) of the most promising isolate using fast protein liquid chromatography (FPLC). In addition, given the reported association between *H. pylori* infection and gastrointestinal carcinogenesis, the cytotoxic activity of the bacteriocin-like protein precipitate obtained from the most promising LAB isolate was evaluated using Caco-2 cells as an exploratory functional assessment alongside its primary antimicrobial and probiotic characterization.

## Methods

### Isolation of lactic acid bacteria (LAB)

#### Sample preparation

LAB were isolated from a total of twelve samples, comprising three buffalo milk samples obtained from a dairy farm and three samples each of natural yogurt, pickle brine, and Karish cheese purchased from local markets. Samples were selected based on their well-established role as traditional fermented and nutrient-rich products known to support the growth and diversity of LAB [15–16]. Samples were transported to the laboratory in sterile containers within cooling bags. Initially, all samples were serially diluted up to 10⁻⁶, where 1 mL of buffalo milk and pickle brine samples were mixed with 9 mL of phosphate-buffered saline (PBS) to obtain a 10⁻¹ dilution, then serially diluted. Following Goraya et al. (2013), one gram of natural yogurt, Karish cheese, and honeycomb was individually suspended in 10 mL of sterile PBS, homogenized by shaking, and serially diluted [17].

Additionally, honeycombs and dead honeybees were gathered from a local apiary as additional isolation sources as honeybees possess a diverse LAB microbiota in their honey crop, which is acquired through the consumption of pollen and nectar and through social interactions within the colony. The honey crop is an important site for microbial colonization and fermentation processes and is recognized as a suitable niche for LAB isolation. Ten dead honeybees were collected for LAB isolation. Surface sterilization was performed by vortexing each bee for few seconds as preliminary cleaning step in a sterile eppendorf tube containing 1 mL of sterile distilled water, followed by immersion in 70% (v/v) ethanol for 1 min. The bees were then rinsed once in sterile distilled water for 1 min to remove residual ethanol prior to further processing. Under aseptic conditions, the honey crop (honey stomach) was dissected from each bee, and each crop was individually crushed in sterile eppendorf tubes containing 1 mL sterile distilled water. Serial ten-fold dilutions were then prepared using sterile PBS [18–19].

### Culturing samples

Using the pour plate method, 1 mL aliquots of serial ten-fold diluted samples (10⁻⁵ and 10⁻⁶) were inoculated onto de Man, Rogosa, and Sharpe (MRS) agar plates (Sigma-Aldrich, USA). These dilutions were selected based on preliminary trials, which showed that lower dilutions resulted in confluent growth, whereas 10⁻⁵ and 10⁻⁶ provided well-isolated colonies suitable for the selection and sub-culturing of individual LAB isolates. The plates were incubated for 48 h at 37 °C under microaerophilic conditions in an anaerobic jar. After incubation, well-isolated colonies with distinct morphology were transferred to sterile MRS broth. Purity was confirmed by streaking onto MRS agar and microscopic examination. All isolates were examined microscopically for cell morphology and Gram staining characteristics. Catalase testing and colony morphology were used as preliminary screening criteria for the selection of presumptive LAB. Only Gram-positive and catalase-negative isolates were retained for further analysis [20].

## Preparation of LAB-cell-free supernatant (CFS)

To prepare CFS, each LAB isolate was inoculated into 10 mL of MRS broth and incubated for 24 h at 37 °C. Cultures were centrifuged at 5000 rpm for 15 min, and the resultant supernatants were neutralized with sterile 0.5 N NaOH. CFSs were sterilized by filtration through a 0.22 μm Millipore membrane syringe filter [21]. The CFS samples were then evaluated for their antibacterial activity against *H. pylori* isolates.

### Screening of anti-*H. pylori* activity of LAB-CFSs

Agar disc diffusion method (Kirby–Bauer style) was used to assess the anti-*H. pylori* activity of LAB-CFSs [22]. CFSs from all LAB isolates were tested against 20 *H. pylori* isolates previously isolated from Egyptian patients, characterized, and categorized into four groups based on their antibiotic susceptibility by Ali et al. (2025) [23]. Briefly, these isolates were recovered from rapid urease test (RUT)-positive antral gastric biopsy specimens, cultured on Columbia blood agar supplemented with 7% sheep blood and selective antibiotics under microaerophilic conditions at 37 °C for 3–10 days, and identified based on colony morphology, Gram staining, urease and catalase activities, with PCR confirmation [23]. Using a sterile cotton swab, an *H. pylori* inoculum (1.5 × 10⁸ CFU/mL) was prepared and spread onto Mueller–Hinton agar (MHA) supplemented with 7% sheep blood. Sterile filter paper discs loaded with 20 µL of each CFS were aseptically placed on the agar surface. Plates were incubated under microaerophilic conditions at 37 °C for 48 h following a 1 h pre-diffusion period at 4 °C allow diffusion of CFS into the agar, as described previously [24]. Zones of inhibition were measured in millimeters (mm) [25–27]. The assay was performed on three independently prepared agar plates for each tested isolate using the same CFS, and the results were expressed as mean ± SE.

### Determination of Minimum Inhibitory Concentration (MIC) and Minimum Bactericidal Concentration (MBC) of the LAB-CFS(s)

The MIC of the most potent LAB-CFS was determined using the micro-broth dilution method according to CLSI (2020) [28]. Mueller–Hinton broth (MHB) (Sigma-Aldrich, USA) supplemented with lysed sheep blood served as the growth medium. A protein-enriched crude precipitate including bacteriocin-like compounds obtained from the LAB-CFS, as described in Sect. 7.1, was used for MIC determination. Stock solutions were prepared at 1500 µg/mL and serially diluted two-fold to obtain eight concentrations ranging from 750 to 5.86 µg/mL. A fresh *H. pylori* inoculum was prepared in sterile 0.85% NaCl and adjusted to 0.5 McFarland turbidity, as described previously for inoculum standardization [29–30] and 10 µL was added to each well. Plates were incubated at 37 °C for 48 h under microaerophilic conditions [31–30]. The MIC was defined as the lowest concentration showing no visible growth. Growth was assessed by visual inspection of turbidity compared with the growth control. Wells showing any detectable turbidity were considered positive for bacterial growth. In cases of weak or partial turbidity, results were interpreted as growth after comparison with positive and negative controls. Positive (inoculum only) and negative (extract only) controls were included in each assay. The MBC was determined by sub-culturing 100 µL from wells showing no visible growth onto MHA supplemented with 7% sheep blood, followed by incubation under microaerophilic conditions at 37 °C for 48 h. The MBC was defined as the lowest concentration at which no bacterial colonies were observed [32].

### Tolerance level

The tolerance level of each CFS was assessed using the MBC/MIC ratio, which classifies the antimicrobial activity of each LAB-CFS as either bacteriostatic (MBC/MIC ratio ≥ 4) or bactericidal (MBC/MIC ratio < 4). This approach provides a straightforward method for determining whether the extract inhibits growth or exerts a killing effect [33].

## Screening of synergistic antibiotic action with LAB-CFSs

The antibiotic adjunct activity of the LAB-CFSs was assessed using the disc diffusion method on MHA plates supplemented with 7% sheep blood inoculated with different *H. pylori* isolates. After placing different antibiotic discs clarithromycin (CLR, 15 µg /disk), metronidazole (MET, 5 µg /disk), amoxicillin (AX, 25 µg /disk), tetracycline (TE, 30 µg /disk), rifampicin (RA, 30 µg /disk) and levofloxacin (LEV, 5 µg /disk), 20 µL of each LAB-CFS was applied to each disc separately. Finally, all the plates were incubated at 37 °C under microaerophilic conditions for 48 h following a 1 h pre-diffusion period at 4 °C. Inhibition zone diameters (mm) were measured and compared to those of the antibiotics and LAB-CFS alone. The assay was performed on three independently prepared agar plates for each tested isolate using the same CFS, and the results were expressed as mean ± SE. Combination effects were classified as synergistic if the inhibition zone of the combination exceeded the sum of the zones of the individual agents, additive if the combination zone equaled the sum, and antagonistic if the combination zone was smaller than the sum of the individual zones ; in addition to an enhanced effect (partial synergy) was recorded when the combined zone exceeded the larger single-agent zones but remained lower than their sum [26, 34].

## Screening of potential probiotic criteria

The most promising LAB isolate was selected for further evaluation of its probiotic criteria based on the results of anti-*H. pylori* activity, MIC/MBC values, and synergistic effect with antibiotics. Probiotic characterization included cell surface traits (hydrophobicity, auto-aggregation, and co-aggregation), safety-related criteria (hemolytic activity and antibiotic resistance), and gastrointestinal survivability (acid and bile tolerance), to assess the isolate’s ability to survive under gastrointestinal conditions. All experiments in this section were conducted in triplicate to ensure reproducibility.

### Bile salt tolerance

Bile salt tolerance was evaluated according to the modified protocols of Manovina et al. (2022) [35] and Amengialue et al. (2024) [36]. One milliliter of the LAB isolate exhibiting the highest anti-*H. pylori* activity, and 1 mL of *Lactiplantibacillus plantarum* ATCC 8014 (reference strain; *L. plantarum*) was used for the assay. Bacterial suspensions were adjusted to OD₆₀₀ = 1 and inoculated into MRS broth supplemented with 0.5%, 1%, 1.5%, and 2% (w/v) bile salts (Oxford Lab Fine Chem LLP, India), a commercially available bile salts mixture and into bile salt–free MRS broth as a control. After incubation at 37 °C for 4 h, bacterial growth was measured at 600 nm. Tolerance was assessed based on the growth in bile salt supplemented media in comparison to the control, and the survival rate was calculated using the following formula:

$$\mathrm{Survival}\;\mathrm{rate}\%=\frac{{\mathrm{OD}}_{4\mathrm h}}{{\mathrm{OD}}_{\mathrm{control}\;4\mathrm h}}\times100$$

Where:

$\:{\mathrm{O}\mathrm{D}}_{4\mathrm{h}}$ = optical density of the culture in the presence of bile salts after 4 h, and $\:{\mathrm{O}\mathrm{D}}_{\mathrm{c}\mathrm{o}\mathrm{n}\mathrm{t}\mathrm{r}\mathrm{o}\mathrm{l}\:4\mathrm{h}}$ = optical density of the culture in bile salt–free MRS broth after 4 h.

For interpretation of bile salt tolerance, survival rates were compared with values commonly reported in the probiotic literature, where survival of approximately 50% or greater in the presence of bile salts is generally considered indicative of acceptable bile tolerance for probiotic candidates [37].

### Acid tolerance

Acid tolerance of LAB isolates was evaluated according to the modified protocols of Li et al. (2020) [38] and Manovina et al. (2022) [35]. One milliliter of the LAB isolate that exhibited the highest anti-*H. pylori* activity, alongside 1 mL of *L. plantarum* ATCC 8014 as the reference strain, was used for the assay. Bacterial suspensions were adjusted to OD₆₀₀ = 1 and inoculated into MRS broth adjusted to pH 1, 2, 3, and 4 using 1 N HCl, with pH 7 serving as the control. All tubes were incubated at 37 °C for 4 h, and bacterial growth was measured at 600 nm using a spectrophotometer. The survival percentage was calculated as follows:

$$\mathrm{Survival}\;\mathrm{rate}\%=\frac{{\mathrm{OD}}_{4\mathrm h}}{{\mathrm{OD}}_{\mathrm{control}\;4\mathrm h}}\times100$$

For interpretation of acid tolerance, survival rates were compared with values commonly reported in the probiotic literature, where survival of approximately 50% or greater under acidic conditions is often considered indicative of acceptable tolerance for probiotic candidates [37].

### Cell surface properties

#### Cell surface hydrophobicity

Cell surface hydrophobicity was evaluated based on bacterial affinity to hydrocarbons, following the method of Panicker et al. (2018) [39], Wang et al. (2018) [40], and Shaheen et al. (2019) [41], with slight modifications. A 24-hour-old culture of the LAB isolate exhibiting the highest anti-*H. pylori* activity and *L. plantarum* ATCC 8014 (reference strain) was prepared in PBS (pH 7.4) and adjusted to an initial absorbance (A₀) of 0.7–0.8 at 600 nm. Three milliliters of the bacterial suspension were mixed separately with 1 mL of either xylene or chloroform, vortexed for 2 min, and incubated at 37 °C for 1 h to allow phase separation. The absorbance of the aqueous phase (A₁) was then measured at 600 nm. Cell surface hydrophobicity (H%) was calculated as:

$$H\%=\left(1-A_1A_0\right)\times100$$

#### Cellular auto-aggregation

Auto-aggregation ability was determined according to Karaseva et al. (2023) [42], with slight modifications. Overnight cultures of the LAB isolate showing the highest antimicrobial activity and *L. plantarum* ATCC 8014 were harvested by centrifugation at 5000 rpm for 15 min, washed twice with sterile PBS (pH 7.4), and resuspended to an initial optical density (A₀) of 0.7–0.8 at 600 nm. The bacterial suspensions were incubated at 37 °C for 4 h without agitation. After incubation, 1 mL of the upper phase was collected, and the absorbance at 600 nm (A₄) was measured. Auto-aggregation percentage was calculated as:

$$\mathrm{AutoA}\left(\%\right)=\left(1-A_4/A_0\right)\times100$$

#### Co-aggregation

Co-aggregation ability was evaluated according to Tuo et al. (2013) [43], with slight modifications. The LAB isolate with the highest antimicrobial activity and *L. plantarum* ATCC 8014 were tested against *Staphylococcus aureus* ATCC 25,923, *Escherichia coli* ATCC 25,922, and *Klebsiella pneumoniae* (identified by Vitek-2, bioMérieux, Marcy l’Etoile, France). These strains were selected as representative Gram-positive and Gram-negative pathogens for evaluation of the co-aggregation ability of the selected LAB isolate. Bacterial cell suspensions were prepared as described for the auto-aggregation assay. Equal volumes (1 mL) of LAB and pathogenic suspensions were mixed, vortexed for 10 s, and incubated at 37 °C for 4 h without agitation. Absorbance at 600 nm was measured for the LAB suspension alone (Aₓ), pathogen suspension alone (A_γ_), and the mixture (A₍ₓ₊_γ_₎) before and after incubation. Co-aggregation percentage was calculated as:

$$\begin{aligned}\mathrm{CoA}(\%)\;&=\;{[(A_X+A_Y)/2-A_{X+Y}]/(A_X+A_Y)/2}\\&\times100\end{aligned}$$

### Assay of safety aspects

#### Hemolysis activity

Hemolytic activity was evaluated according to **Katiku** et al. **(2022)** [44], with slight modifications. Overnight cultures of the LAB isolate exhibiting the highest antimicrobial activity and *L. plantarum* ATCC 8014 were streaked onto MRS agar supplemented with 5% sheep blood and incubated at 37 °C for 48 h. Plates were examined for hemolysis and classified as α-hemolysis (partial, greenish zone), β-hemolysis (complete, clear zone), or γ-hemolysis (no hemolysis). *L. plantarum* ATCC 8014 was used as the γ-hemolysis control.

#### Antibiotic sensitivity test

Antibiotic susceptibility was determined using the Kirby–Bauer disc diffusion method according to CLSI (2020) guidelines [28]. An overnight culture of the LAB isolate exhibiting the highest antimicrobial activity was adjusted to 0.5 McFarland standard (~ 10⁸ CFU/mL) and spread onto MRS agar plates (Sigma-Aldrich, USA). Antibiotic discs of tetracycline (TE, 30 µg), clindamycin (DA, 2 µg), azithromycin (AZM, 15 µg), amoxicillin (AX, 25 µg), levofloxacin (LEV, 5 µg), rifampicin (RA, 30 µg), cephradine (CE, 30 µg), and metronidazole (MET, 5 µg) were applied. These antibiotics were selected to represent clinically relevant antimicrobial classes commonly used in human medicine and *H. pylori* eradication therapy. Plates were incubated at 37 °C for 24 h, and inhibition zone diameters were measured in millimeters. The experiment was performed in triplicate, and results were expressed as mean ± SE. As standardized clinical breakpoints are not available for *Lactobacillus* spp., inhibition zones were interpreted using LAB-specific disc diffusion criteria reported by Charteris et al. (1998 ) [45] and Sharma et al. (2017) [46], where applicable: resistant (R) ≤ 14 mm, intermediate (I) 15–19 mm, and susceptible (S) ≥ 20 mm. This interpretation is intended for preliminary safety assessment and not for clinical susceptibility classification.

## Statistical analysis

A one-way ANOVA was performed using Statistical Package for the Social Sciences (SPSS) software (version 23.0). The analysis was applied to inhibition zone diameters and probiotic characterization parameters (including hydrophobicity and aggregation assays), as appropriate. Comparisons were made among LAB isolates and their combinations with antibiotics. When significant differences were observed (*p* < 0.05), post hoc multiple comparison tests were performed to determine pairwise differences between groups.

## Molecular identification of LAB isolate by 16 S rRNA gene sequencing

### Genomic DNA extraction

Genomic DNA was extracted using the QIAamp DNA Mini Kit (Qiagen, Germany) following the manufacturer’s instructions. Briefly, 200 µL of ATL buffer and 20 µL of proteinase K were added to 200 µL of the sample to lyse the cells, and the mixture was incubated at 56 °C for 10 min. To facilitate DNA binding to the QIAamp Mini Spin Column, 200 µL of 96% ethanol was added, and the mixture was vortexed for 15 s. The lysate was then transferred to a QIAamp Mini spin column placed in a 2 mL collection tube and centrifuged at 6,000 × g (8,000 rpm) for 1 min. DNA adhered to the silica membrane of the column, and three consecutive washing steps were performed using the buffers provided in the kit. Purified DNA was eluted and used as the template for PCR amplification.

### PCR amplification, sequencing, and phylogenetic analysis

The V4 region of the 16 S rRNA gene was amplified using the primer pair: Forward (F) WLAB1: TCCGGATTTATTGGGCGTAAAGCGA, Reverse (R) WLAB2: TCGAATTAAACCACATGCTCCA. PCR was performed according to Kim et al. (2015) [47], with the following conditions: initial denaturation at 94 °C for 5 min; 35 cycles of denaturation at 94 °C for 30 s, annealing at 60 °C for 40 s, and extension at 72 °C for 45 s; followed by a final extension at 72 °C for 10 min. PCR products were visualized on a 2% agarose gel containing ethidium bromide alongside a 100 bp DNA ladder. Negative and positive controls were included in each assay. After approximately 30 min of electrophoresis, the gel was observed under UV light and documented using a gel documentation system. The presence of clear amplicons of the expected size confirmed the integrity and suitability of the extracted genomic DNA for downstream sequencing analysis. PCR products were purified using the QIAquick PCR Product Extraction Kit (Qiagen, Valencia). Sequencing reactions were performed using the BigDye Terminator v3.1 Cycle Sequencing Kit (Perkin-Elmer) and purified with a Centri-Sep spin column. DNA sequences were obtained using an Applied Biosystems 3130 Genetic Analyzer (HITACHI, Japan).

The resultant sequences were identified using Basic Local Alignment Search Tool (BLAST) at the National Center for Biotechnology Information (NCBI) [48]. Multiple sequence alignment was performed using MEGA 11, and closely related sequences were retrieved from GenBank for phylogenetic analysis. A phylogenetic tree was constructed using the neighbor-joining method and visualized with the iTOL online tool [49]. The sequence was submitted to GenBank using the BankIt submission tool to obtain unique accession numbers.

## Chemical analysis of the most potent LAB-CFS

### Purification of a putative bacteriocin-like antimicrobial compound

One milliliter of the LAB isolate exhibiting the highest activity against the tested *H. pylori* isolates was inoculated into 200 mL of MRS broth and incubated overnight at 37 °C. Cells were then removed by centrifugation at 6,000 rpm for 15 min, and the supernatant was filtered through a 0.22 μm syringe filter to obtain a CFS. The CFS was subjected to acetone precipitation by adding four volumes of cold acetone, followed by incubation at − 28 °C overnight. The resulting precipitate was collected, residual acetone was allowed to evaporate, and the protein-enriched crude precipitate was weighed. Approximately 0.3 g of crude precipitate was obtained from 200 mL of CFS and resuspended in PBS. This protein-enriched crude precipitate, containing a putative bacteriocin-like compounds, was used for MIC determination, cytotoxicity testing, and further purification by FPLC, followed by SDS-PAGE analysis of the active fraction [50–51].

### Cytotoxic activity of LAB protein precipitate against Caco-2 cells

The cytotoxic activity of the LAB protein precipitate containing a putative bacteriocin-like compounds was evaluated against Caco-2 cells using 3-(4,5-dimethylthiazol-2-yl)-2,5-diphenyltetrazolium bromide (MTT) assay [52]. The Caco-2 cell line (ATCC^®^ HTB-37™; American Type Culture Collection, Manassas, VA, USA) was selected as a well-established in vitro model of human colorectal adenocarcinoma for evaluating the biological activity of the tested preparation, particularly in light of the reported association between *H. pylori* infection and gastrointestinal carcinogenesis [76]. The assay was performed at Science Way Company, Cairo, Egypt. Briefly, Caco-2 cells (1 × 10⁵ cells/mL) were seeded into 96-well plates and incubated for 24 h to allow monolayer formation. The cells were then treated with two-fold serial dilutions of the LAB protein precipitate prepared in Roswell Park Memorial Institute (RPMI) medium supplemented with 2% serum, while untreated cells served as the negative control. Following incubation, MTT reagent was added and the plates were incubated for 4 h. The formed formazan crystals were dissolved in dimethyl sulfoxide (DMSO), and cell viability was determined by measuring the optical density at 560 nm with background correction at 620 nm. The results were expressed as IC₅₀ values, defined as the concentration required to inhibit 50% of cell growth, as calculated from the dose–response curve.

###  Fast Protein Liquid Chromatography (FPLC)

FPLC was performed to separate proteinaceous components of the precipitate and identify fractions associated with antimicrobial activity. The system (ÄKTA Avant 150, GE Healthcare Life Sciences) was used at the Central Laboratories Network, National Research Centre, Cairo, Egypt. Protein samples were mixed with binding buffer A (50 mmol/L sodium phosphate buffer, pH 6.0) and applied to an SP Sepharose Fast Flow column using the ÄKTA Avant system. Bound proteins were eluted with a linear gradient of buffer B (1 mol/L NaCl) at a flow rate of 2 mL/min. Elution was monitored using a UV detector at 220 and 280 nm.

Collected fractions were tested for antibacterial activity using the agar well diffusion method. MHA plates supplemented with 7% sheep blood were inoculated with *H. pylori* isolates using sterile cotton swabs. Wells (7 mm diameter) were cut into the agar, and 100 µL of each fraction was added. Plates were left at 4 °C for a 1 h diffusion period, then incubated at 37 °C for 24 h. The diameter of inhibition zones (mm) was measured to identify active fractions [53].

### Sodium Dodecyl Sulfate–Polyacrylamide Gel Electrophoresis (SDS-PAGE)

Active fractions obtained from FPLC purification of LAB proteins precipitate containing a putative bacteriocin like compounds were analyzed by SDS-PAGE. Samples were denatured in 2× SDS sample buffer (0.25 M Tris-HCl, pH 6.8; 10% SDS; 20% glycerol; 10% 2-mercaptoethanol) by boiling for 90 s and cooled on ice prior to loading. Electrophoresis was performed using a discontinuous system with a 10% resolving gel and 4% stacking gel prepared with acrylamide/bis-acrylamide and polymerized using Ammonium persulfate (APS) and Tetramethylethylenediamine (TEMED). Gels were run in Tris-glycine-SDS buffer (25 mM Tris, 192 mM glycine, 0.1% SDS, pH 8.3) at 50 V until samples entered the resolving gel, then at ~ 100 V until completion. Proteins were visualized by staining with 1% Coomassie Brilliant Blue R-250 and destaining in methanol-acetic acid solution until clear bands were observed [54].

## Results

## Isolation of LAB isolates

**Fig. 1:**
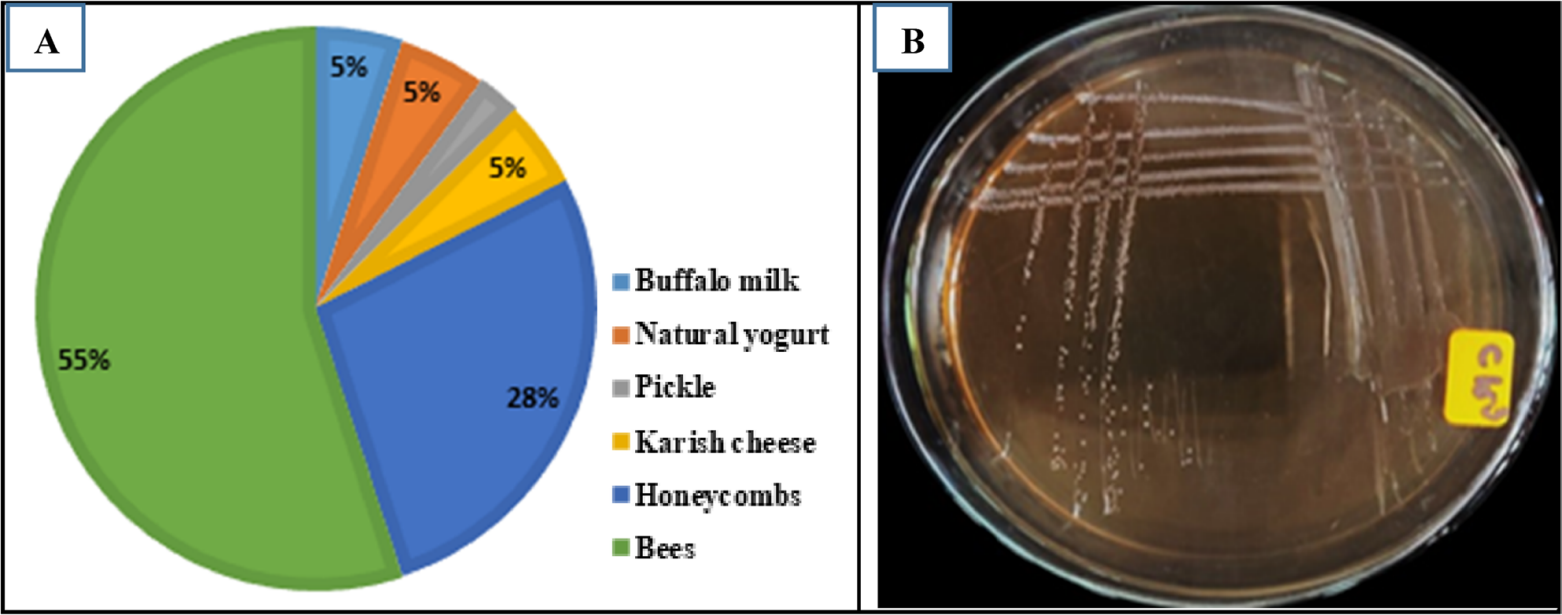
**A**. The percentage of LAB obtained from different isolation sources. **B**. Small, white to creamy colonies typical of LAB on MRS medium

Forty LAB isolates were obtained from different isolation sources at different frequencies, as shown in Fig. 1A. The highest number of LAB bacterial isolates was obtained from the honeybees, followed by the honeybee combs. Upon colony morphological examination, all the pure isolates were characterized by a small white/creamy colony appearance, as shown in Fig. 1B. All colonies were catalase negative. Upon microscopic examination, they were all rod-shaped and Gram-positive. After purification, CFS for each isolate was prepared, neutralized, and sterilized for subsequent testing against *H. pylori* isolates.

### Screening of anti-*H. pylori*activity of LAB-CFSs

**Fig. 2:**
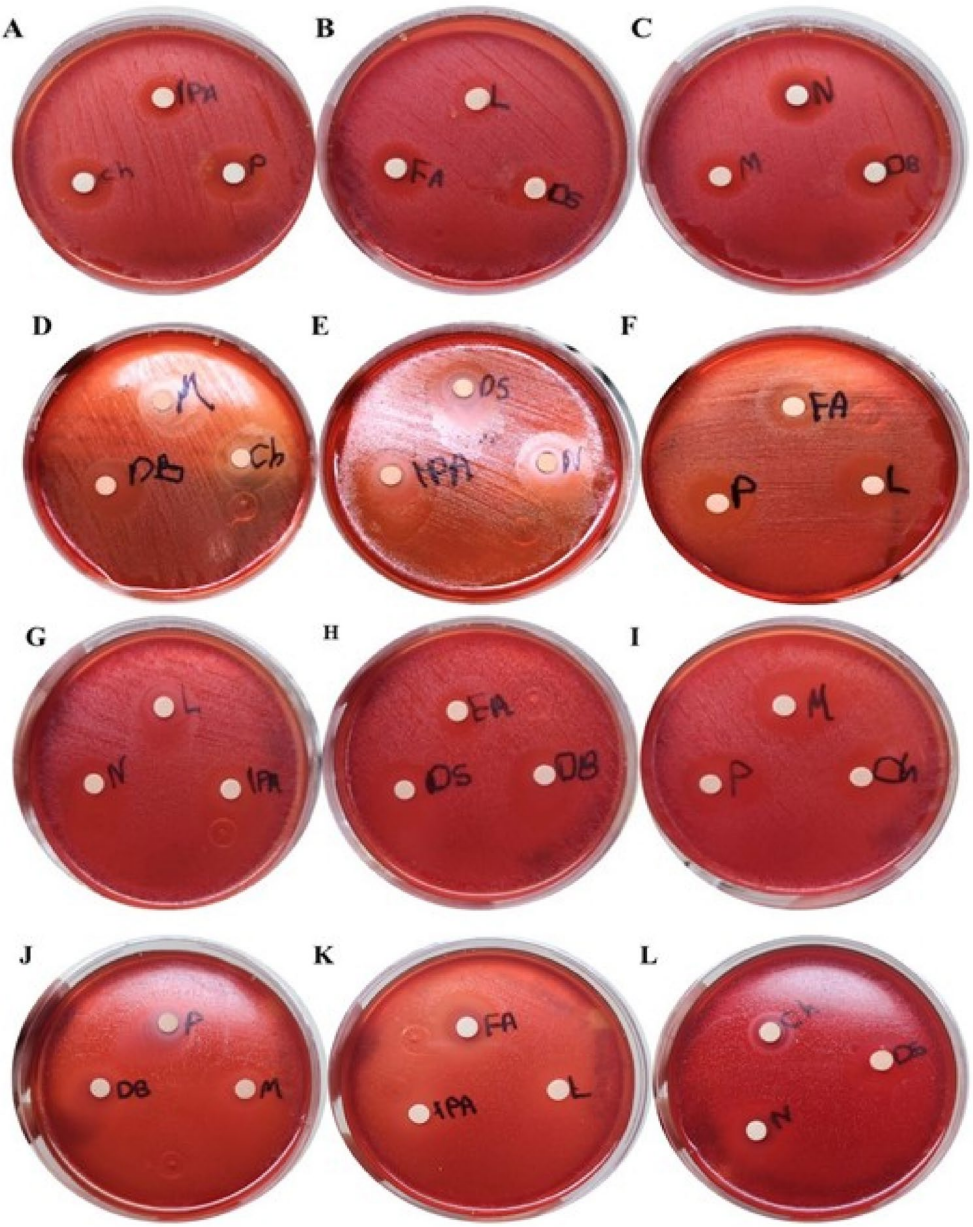
Anti-*H. pylori* activity of nine LAB-CFSs assessed using the disc diffusion method against four representative isolates. Panels A–C correspond to group 1, panels D–F to group 2, panels G–I to group 3, and panels J–L to group 4

Of the 40 LAB isolates screened, nine CFS exhibited anti-*H. pylori* activity. These isolates designated as N, L, 1PA, P, M, C, FA, DB, and DS exhibited measurable inhibitory effects against all tested *H. pylori* isolates, results were represented as the mean of each group, as shown in Fig. 2 and Table 1. Overall, the LAB isolates displayed relatively similar activity against each group, with mean inhibition zone diameters ranging from 12.41 mm for isolate L (the smallest zone) to 15.74 mm for isolate N (the largest zone). The effect of LAB-CFSs on the tested parameters was statistically significant (*p* < 0.05), confirming meaningful differences in antimicrobial activity among the tested isolates as shown in Table 1.

**Table 1:** Comparative mean inhibition zone diameters (mm) of nine LAB-CFSs against the different *H. pylori* groups, highlighting isolate N as the most active

H. pylori groups	Mean of inhibition zone diameter in mm (± SE)								
	*N*	L	1PA	*P*	M	C	FA	DB	DS
1	15.66 ± 0.33	10 ± 0.00	13 ± 0.00	16 ± 0.00	13 ± 0.00	15.66 ± 0.33	15.33 ± 0.33	15.33 ± 0.33	13 ± 0.00
2	15 ± 0.00	14.66 ± 0.33	15.33 ± 0.33	14.66 ± 0.33	15.66 ± 0.33	16.33 ± 0.33	14 ± 0.00	15.66 ± 0.33	16.66 ± 0.33
3	16.66 ± 0.33	15 ± 0.00	16.66 ± 0.33	15.33 ± 0.33	16.66 ± 0.66	15.66 ± 0.33	13 ± 0.00	16 ± 0.00	19 ± 1.15
4	15.66 ± 0.33	10 ± 0.00	13.33 ± 0.33	15.66 ± 0.33	14 ± 0.00	14.66 ± 0.33	17 ± 0.00	15.66 ± 0.33	13 ± 0.00
Mean	**15.74 ± 0.43**	15.66 ± 1.39	15.57 ± 0.86	15.41 ± 0.28	15.41 ± 0.82	14.83 ± 0.34	14.83 ± 0.86	14.58 ± 0.13	12.41 ± 1.47
*P*-value	0.000	0.000	0.000	0.000	0.000	0.000	0.000	0.000	0.000

### Determination of MIC and MBC

The LAB-CFSs exhibited variable MIC values across the different *H. pylori* groups. Among them, the P and DB CFSs were the most effective against group one isolates, exhibiting the lowest MIC values (187.5 µg/mL). The activity against group four isolates was generally consistent, except for the FA CFS, which recorded a MIC of 750 µg/mL, twice that of the other extracts, indicating weaker inhibitory potency. The most promising CFS was N, which exhibited the lowest MIC value (93.7 µg/mL) against isolates belonging to groups two and three as shown in Table 2.

Regarding bactericidal activity, isolate N demonstrated the highest bactericidal efficacy, with MBC values of 93.7 µg/mL and 187.5 µg/mL against group three and group two isolates, respectively. The remaining samples (L, 1PA, P, FA, DB, and DS) showed intermediate activity, with MBC values mostly ranging from 375 to 750 µg/mL depending on the isolate group. Samples M and C exhibited the weakest bactericidal activity, with MBC values of 750 µg/mL against most tested isolates **(**Table 2**)**.

### Tolerance Level

Assessment of *H. pylori* tolerance levels using MBC/MIC ratios **(**Table 2**)** revealed that most LAB CFSs exhibited strong bactericidal activity against the tested isolates. Exceptions were observed for 1PA against group three and DB against group two isolates, where the MBC/MIC ratio reached 4. These higher ratios indicate reduced bactericidal efficacy compared with the other tested CFSs.

**Table 2:** MIC, MBC, and MBC/MIC ratio values of protein-enriched crude precipitates from different LAB isolates against four groups of *H. pylori* isolates

H. pylori group	Isolate	MIC (µg/mL)	MBC (µg/mL)	MBC/MIC ratio
Gp.1	N	375	375	1
Gp.1	L	375	375	1
Gp.1	1PA	375	750	2
Gp.1	P	187.5	375	2
Gp.1	M	375	750	2
Gp.1	C	375	750	2
Gp.1	FA	750	750	1
Gp.1	DB	187.5	375	2
Gp.1	DS	375	750	2
Gp.2	N	93.7	187.5	2
Gp.2	L	375	375	1
Gp.2	1PA	375	375	1
Gp.2	P	375	750	2
Gp.2	M	750	750	1
Gp.2	C	750	750	1
Gp.2	FA	375	375	1
Gp.2	DB	187.5	750	4
Gp.2	DS	375	375	1
Gp.3	N	93.7	93.7	1
Gp.3	L	750	750	1
Gp.3	1PA	187.5	750	4
Gp.3	P	187.5	187.5	1
Gp.3	M	375	750	2
Gp.3	C	375	750	2
Gp.3	FA	750	750	1
Gp.3	DB	375	750	2
Gp.3	DS	750	750	1
Gp.4	N	375	375	1
Gp.4	L	375	750	2
Gp.4	1PA	375	375	1
Gp.4	P	375	375	1
Gp.4	M	375	750	2
Gp.4	C	375	750	2
Gp.4	FA	750	750	1
Gp.4	DB	375	375	1
Gp.4	DS	375	375	1

## Screening of synergistic antibiotic action with LAB-CFSs

**Fig. 3:**
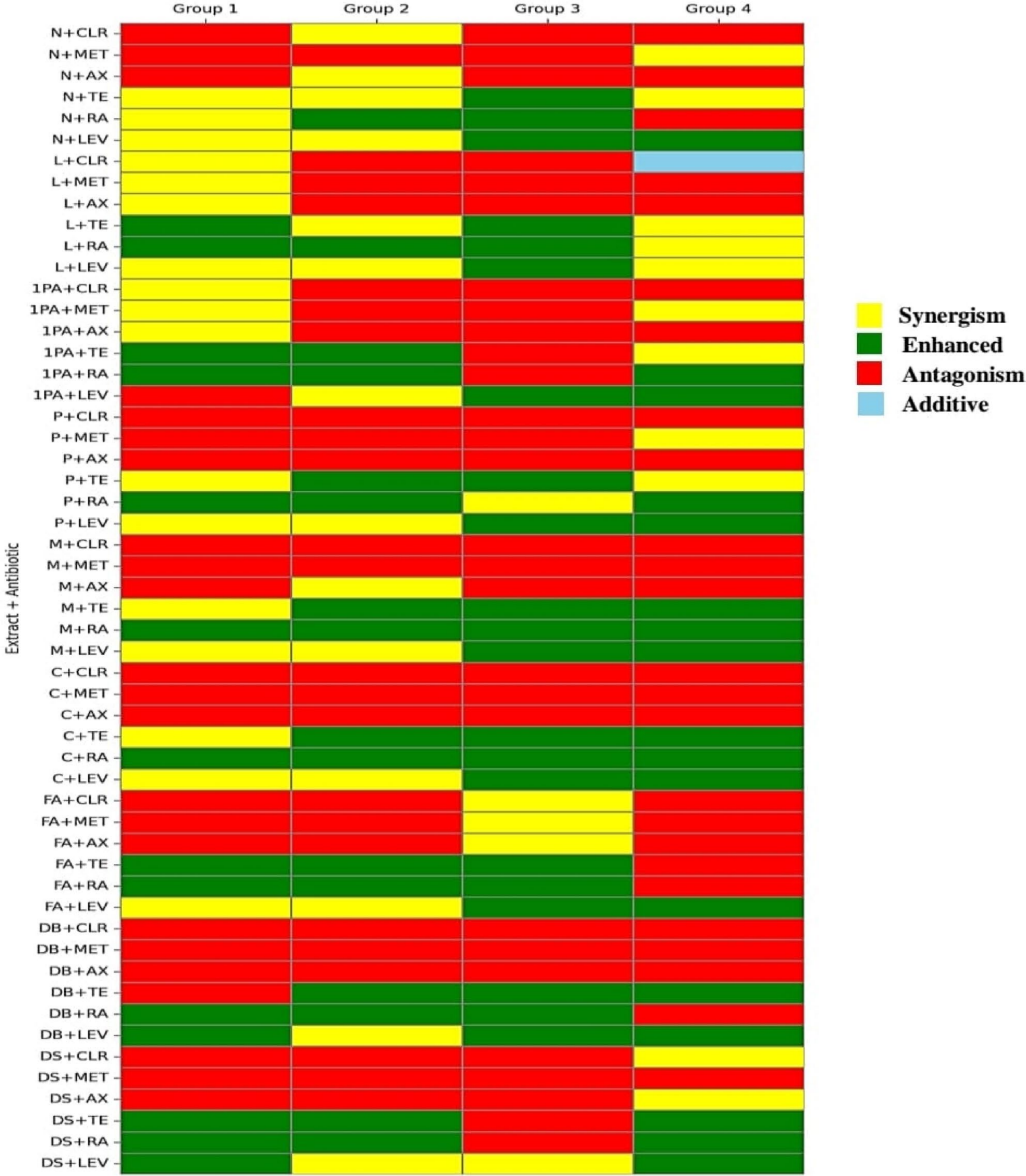
Heat map illustrating the interaction patterns between LAB-CFSs and antibiotics against four *H. pylori* resistance groups. Colors indicates the type of interaction observed: synergistic (combined inhibition zone greater than the sum of individual zones), antagonistic (combined zone smaller than individual zones), additive (combined zone equal to the sum), and enhanced or partial synergy (combined zone larger than the most active single agent but less than the sum). The map highlights variability in responses based on both the LAB isolate and the antibiotic tested

Based on the antibiotic sensitivity profiles of *H. pylori* as shown in Fig S1, and the inhibitory effect of different CFSs against *H. pylori* groups (Table 1), the combined action of LAB-CFSs with antibiotics found to produce variable outcomes depending on both CFS and the tested *H. pylori* group, ranging from antagonism to strong synergy (Fig. 3). For example, DB CFS consistently antagonized CLR, MET, and AX across all groups, and TE in group 1; however, combinations with TE in other groups enhanced activity. DB + RA displayed mutual potentiation across most groups, while DB + LEV showed the strongest up to a 1.9-fold increase (against group 2). Similarly, C-CFS showed antagonism with CLR, MET, and AX but exhibited enhanced or synergistic activity when combined with RA, TE, or LEV, depending upon the tested group. DS-CFS demonstrated antagonism with MET, CLR, and AX in most groups, with synergism mainly observed in combinations with LEV. FA-CFS lost activity when combined with CLR, MET, and AX but produced enhanced or synergistic effects with TE, RA, and LEV especially FA + LEV, which yielded 1.26–2.14-fold increases. M-CFS activity was generally inhibited by CLR, MET, and AX; however, synergistic activity emerged in combinations with AX, TE, RA, and LEV, with M + LEV showing 1.29–2.15-fold increases against groups 1 and 2 (Fig. S2–S6; Tables S1–S5).

**Fig. 4:**
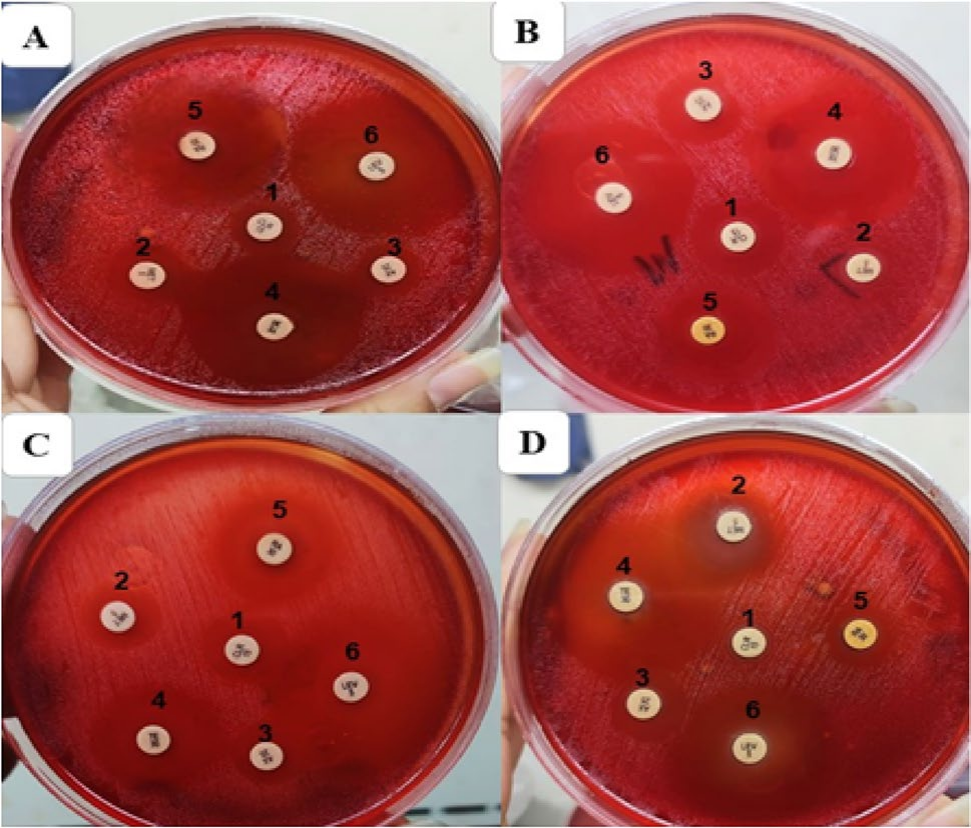
Combination effects of N-CFS with six antibiotics against four *H. pylori* resistance groups. Panels A–D correspond to groups 1–4, respectively. Numbers (1–6) indicate antibiotics: 1 = clarithromycin (CLR, 15 µg); 2 = metronidazole (MET, 5 µg); 3 = amoxicillin (AX, 25 µg); 4 = tetracycline (TE, 30 µg); 5 = rifampicin (RA, 30 µg); 6 = levofloxacin (LEV, 5 µg). The figure illustrates variations in inhibition zone diameters, highlighting synergistic and enhanced interactions

IPA-CFS and P-CFS exhibited similar profiles, showing antagonism with CLR, MET, and AX, but enhanced or synergistic effects with TE, RA, and LEV, depending on the tested group. Where P + LEV produced 1.12–2.1-fold activity increases, particularly against groups 1 and 2. L-CFS showed antagonism with MET, AX, and CLR in several groups, whereas combinations with RA, TE, and LEV enhanced or synergized activity; L + LEV produced 1–2.1-fold increases (Fig. S7–S9; Tables S6–S8). Among them, isolate N consistently showed higher fold increases in antimicrobial activity compared to other CFSs, particularly in combination with levofloxacin (LEV), tetracycline (TE), and rifampicin (RA), indicating comparatively stronger interaction effects across the tested groups (Fig. 4; Table S9). All tested LAB-CFSs and their combinations with antibiotics exhibited statistically significant effects against *H. pylori* isolates (*p* < 0.05) as shown in Table S10 in supplementary file.

## Screening of potential probiotic criteria for the LAB isolate with the highest activity

N-CFS exhibited the highest anti-*H. pylori* activity, both alone and in combination with different antibiotics; therefore, it was selected for further evaluation of its probiotic properties alongside molecular identification.

### Acid tolerance and bile tolerance

**Fig. 5:**
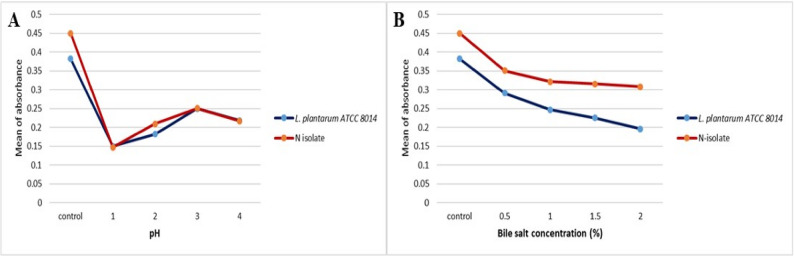
Acid tolerance **(A)** and bile salt tolerance **(B)** of isolate N compared with *L. plantarum* ATCC 8014 after 4 h of exposure to different pH values (1–4) and bile salt concentrations (0.5–2%), respectively. The line graphs show bacterial survival rates, where optical density at 600 nm (OD₆₀₀) reflects bacterial growth intensity. Error bars represent the standard error (0.001–0.002 for acid tolerance and 0.001–0.003 for bile salt tolerance)

The acid tolerance of isolate N and the reference strain *L. plantarum* ATCC 8014 was evaluated by determining their survival rates after 4 h of exposure to acidic conditions (pH 1–4). As shown in Table S11, both strains exhibited varying degrees of acid tolerance. At pH 1, survival was lowest, reaching 32.66% for isolate N and 39.26% for the reference strain. Survival increased with increasing pH, reaching 46.66% and 47.90% at pH 2, and 55.77% and 65.70% at pH 3 for isolate N and the reference strain, respectively. At pH 4, survival rates were 48.22% for isolate N and 57.32% for the reference strain. Overall, isolate N exhibited good acid tolerance, particularly at pH values of 2–4 (Fig. 5), suggesting its potential survival under gastric conditions. Notably, the survival rate at pH 3 exceeded 50%, a value commonly reported as acceptable for preliminary probiotic screening. The bile tolerance of isolate N and the reference strain *L. plantarum* ATCC 8014 was assessed by measuring survival rates after 4 h of exposure to different bile salt concentrations (0.5–2%). As shown in Table S11, isolate N exhibited high survival across all tested concentrations, with survival rates of 78.00%, 71.55%, 70.22%, and 68.44% at 0.5%, 1.0%, 1.5%, and 2.0% bile salts, respectively. In comparison, the reference strain showed survival rates of 76.17%, 64.65%, 58.90%, and 51.30% under the corresponding conditions **(**Fig. 5**)**. Although both strains demonstrated bile tolerance, isolate N maintained greater viability at higher bile concentrations. Furthermore, survival remained above 50% at all tested bile salt concentrations, indicating strong bile tolerance and supporting its potential ability to survive gastrointestinal transit.

### Cell surface properties

**Fig. 6:**
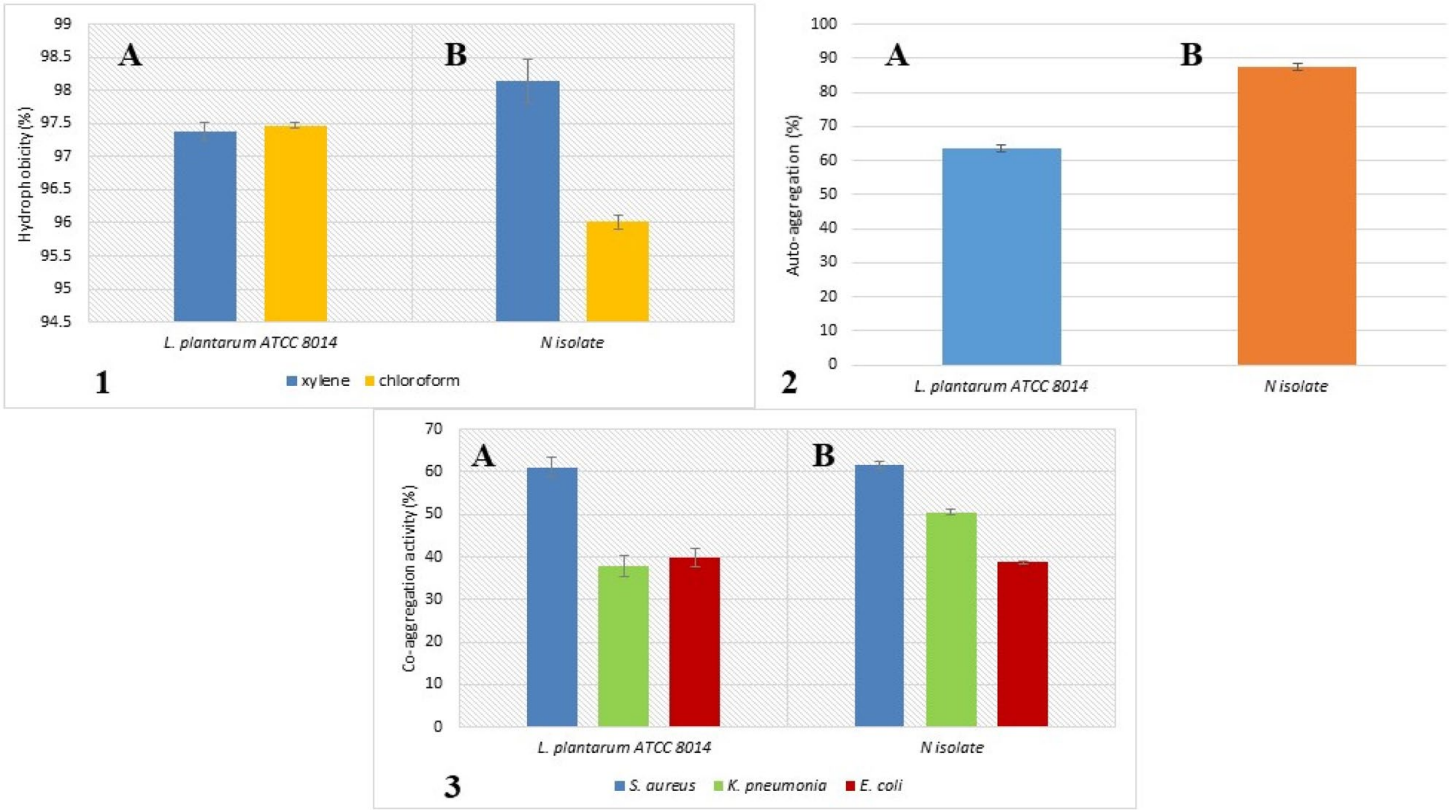
Cell surface properties of LAB isolates. Panel **A** represents the reference strain *L. plantarum* ATCC 8014, and Panel **B** represents isolate N. Subfigure 1 shows cell surface hydrophobicity, Subfigure 2 shows auto-aggregation ability, and Subfigure 3 shows co-aggregation with selected pathogens

Cell surface properties, including hydrophobicity, auto-aggregation, and co-aggregation, were evaluated for isolate N alongside the reference strain *L. plantarum* ATCC 8014. Hydrophobicity was determined by microbial adhesion to hydrocarbons using xylene and chloroform as solvents. Isolate N exhibited high hydrophobicity with both solvents -xylene (98.15 ± 0.57%) and chloroform (96.01 ± 0.19%) while the reference strain recorded 97.38 ± 0.13% and 98.15 ± 0.33%, respectively. Auto-aggregation analysis revealed that isolate N showed higher activity (87.64 ± 0.82%) compared to the reference strain (63.45 ± 0.66%). Co-aggregation ability was assessed against three pathogenic bacteria, *S. aureus* ATCC 25,923, *E. coli* ATCC 25,922, and *K. pneumonia*. Isolate N showed co-aggregation rates of 61.69 ± 1.39%, 38.80 ± 0.58%, and 50.53 ± 1.37% respectively, compared to the reference strain, which recorded 61.69 ± 0.80%, 39.79 ± 2.23% and 37.82 ± 2.42% for the same pathogens as shown in Fig. 6.

### Assay of safety aspects

#### Hemolysis activity

The hemolytic activity testing showed that the isolate N, just like the reference strain, is γ-hemolytic after 48 h of incubation, with neither a clear transparent nor a greenish zone on the streaked area of the blood agar plates (**Fig. S10).**

#### Antibiotic sensitivity test

**Fig. 7:**
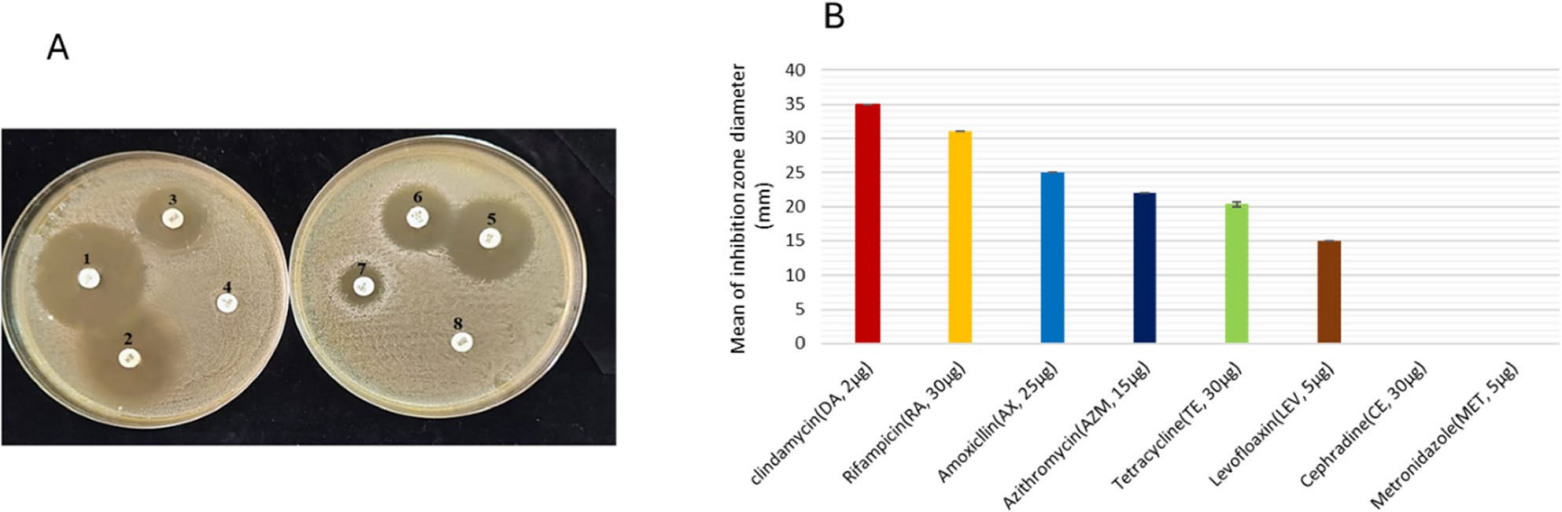
Antibiotic sensitivity pattern of isolate N. (**A**) Disc diffusion assay performed on Mueller–Hinton agar (MHA) showing inhibition zones for eight antibiotics: (1) Clindamycin (DA, 2 µg), (2) Rifampicin (RA, 30 µg), (3) Tetracycline (TE, 30 µg), (4) Metronidazole (MET, 5 µg), (5) Amoxicillin (AX, 25 µg), (6) Azithromycin (AZM, 15 µg), (7) Levofloxacin (LEV, 5 µg), and (8) Cephradine (CE, 30 µg). The original full-length unprocessed image corresponding to this figure is provided in Supplementary Fig. S11. (**B**) Histogram representing the mean inhibition zone diameters (mm ± SE) of isolate N against the tested antibiotics

The antibiotic susceptibility profile of isolate N revealed sensitivity to six out of the eight tested antibiotics, with inhibition zone diameters ranging from 20.33 ± 0.00 mm to 35.00 ± 0.00 mm. The isolate was resistant only to cephradine (CE, 30 µg) and metronidazole (MET, 5 µg), as shown in Fig. 7.

## Molecular identification of the probiotic isolate N

**Fig. 8:**
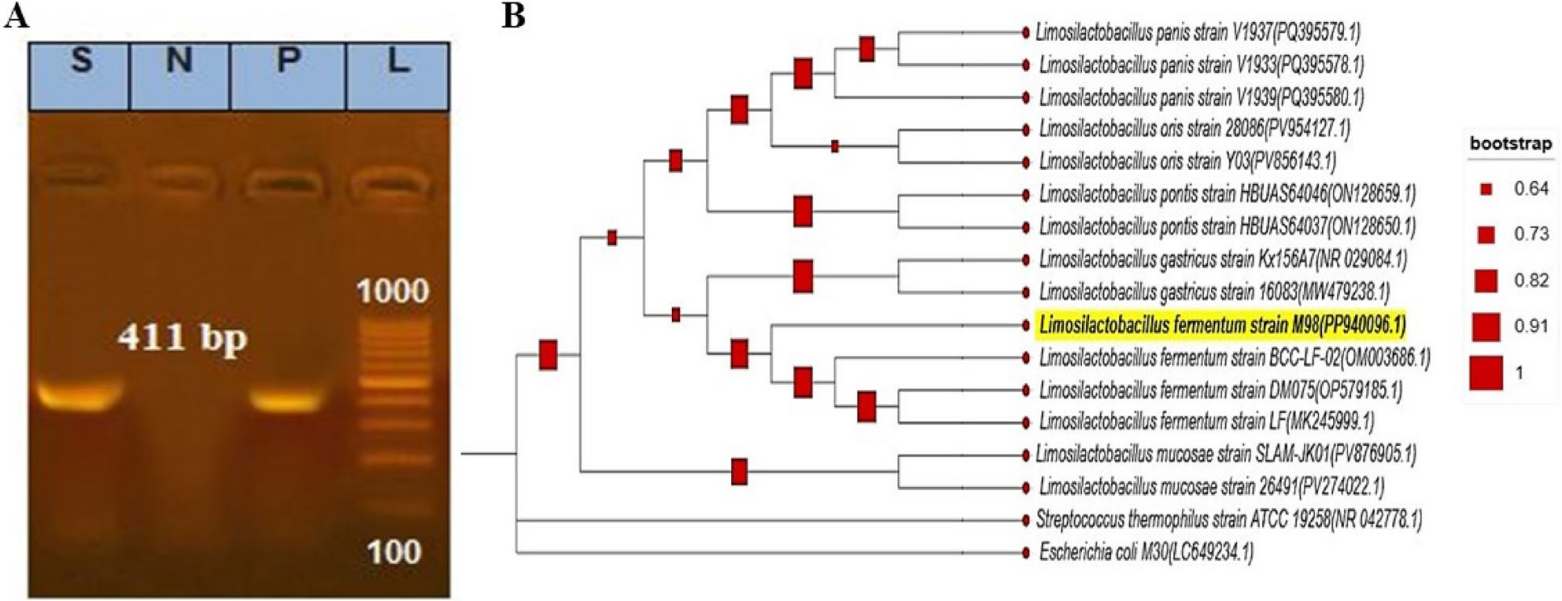
Molecular identification of Isolate N. (**A**) Agarose gel electrophoresis of PCR products of LAB. The 411 bp fragment was detected in the sample. L: molecular weight marker (100 bp ladder). P: positive control. N: negative control. S: sample. The original full-length unprocessed gel image corresponding to this figure is provided in Supplementary Fig. S12 (**B**) Neighbor-joining phylogenetic tree based on 16 S rRNA gene sequences, constructed by Mega 11 and visualized by iTOL online tool, confirming isolate N (*L. fermentum* strain M98, PP940096) clustering with *L. fermentum* strains within the genus *Limosilactobacillus*

The most promising LAB isolate, designated N, was identified using 16 S rRNA gene sequence analysis. A 411 bp PCR amplicon was obtained and visualized by agarose gel electrophoresis **(**Fig. 8**)**. The purified PCR product was sequenced, yielding a high-quality partial 16 S rRNA gene sequence of 393 bp after trimming of low-quality ends. The sequence was analyzed using the BLAST tool (NCBI), which revealed 99.7% similarity with *Limosilactobacillus fermentum (L. fermentum)*. The sequence was subsequently submitted to GenBank under accession number PP940096 as *L. fermentum* strain M98.

To confirm taxonomic placement, a neighbor-joining phylogenetic tree was constructed using MEGA 11 software, incorporating related members of the genus *Limosilactobacillus*. *Streptococcus thermophilus* ATCC 19,258 and *Escherichia coli* M30 were used as outgroups. Phylogenetic analysis showed that isolate N clustered within the genus *Limosilactobacillus* and grouped specifically with *L. fermentum* strains **(**Fig. 8**)**. The obtained partial 16 S rRNA gene sequence (393 bp) covered the V4 hypervariable region, which provides sufficient resolution for reliable genus-level and, in most cases, species-level identification based on comparison with reference sequences in the NCBI database.

## Cytotoxic effect of LAB-precipitated proteins containing putative bacteriocin-like compounds

Microscopic examination of Caco-2 cells treated with LAB-precipitated proteins containing a putative bacteriocin revealed distinct morphological changes indicative of anticancer activity. Cells exposed to higher concentrations exhibited features such as rounding, shrinkage, granulation, and partial detachment from the monolayer, compared to the untreated control, which maintained a normal epithelial appearance (Fig. S13).

**Fig. 9:**
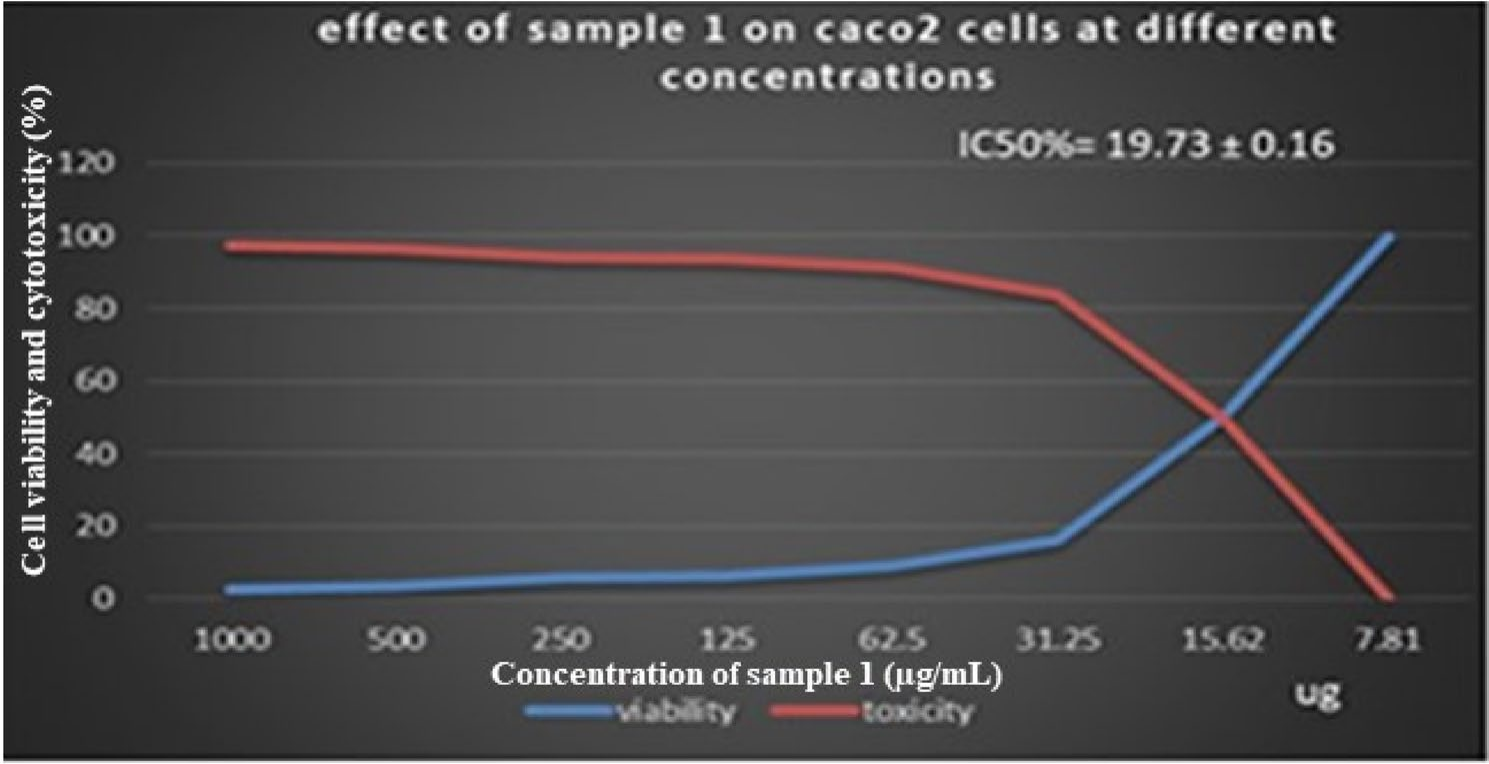
Dose-dependent cytotoxic effect of the protein precipitate containing a putative bacteriocin derived from *L. fermentum* strain M98 on Caco-2 cells following treatment with different concentrations. The line graph shows the percentage inhibition and corresponding cell viability observed at the tested concentrations

Quantitative analysis using the MTT assay confirmed these observations, showing a dose-dependent decrease in cell viability following 24-hour treatment with LAB-precipitated proteins. The highest tested concentration (1000 µg/mL) resulted in 2.6% cell viability, while the lowest concentration (7.81 µg/mL) maintained viability above 99%. The calculated IC₅₀ value was 19.73 ± 0.16 µg/mL indicating a significant reduction in Caco-2 cell viability following treatment with the LAB-precipitated proteins containing a putative bacteriocin, as shown in Fig. 9.

## Fast Protein Liquid Chromatography (FPLC)

**Fig. 10:**
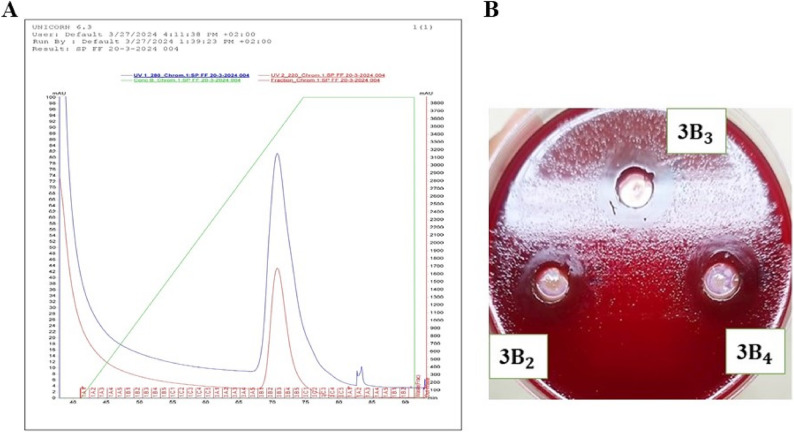
(**A**) FPLC chromatogram showing two peaks: the first peak (red) at 220 nm and the second peak (blue) at 280 nm, corresponding to fractions 2, 3, and 4. The original full-length unprocessed FPLC image corresponding to this figure is provided in Supplementary Fig. S14. (**B**) Anti-*H. pylori* activity of the same fractions

FPLC was applied to the crude protein precipitate containing putative bacteriocin-like compounds from *L. fermentum* for the separation and purification of bioactive fractions. A total of 22 fractions were collected, among which three successive fractions (3B₂, 3B₃, and 3B₄) exhibited distinct absorbance peaks at 220 nm and 280 nm, corresponding to peptide bonds and aromatic amino acids, respectively. These fractions were eluted between 70 and 75 min at a flow rate of 2 mL/min. The antimicrobial activity of the fractions was evaluated against *H. pylori*. All fractions showed inhibitory effects, with fraction 3B₃ demonstrating the strongest activity, producing a mean inhibition zone diameter of 23 ± 0.00 mm. Fractions 3B₂ and 3B₄ produced inhibition zones of 19.33 ± 0.33 mm and 20.33 ± 0.33 mm, respectively (Fig. 10**)**. These results suggest that fraction 3B₃ contains the highest concentration of the bioactive bacteriocin-like compound.

## 8. SDS-PAGE analysis of the active bacteriocin-like fraction

**Fig. 11:**
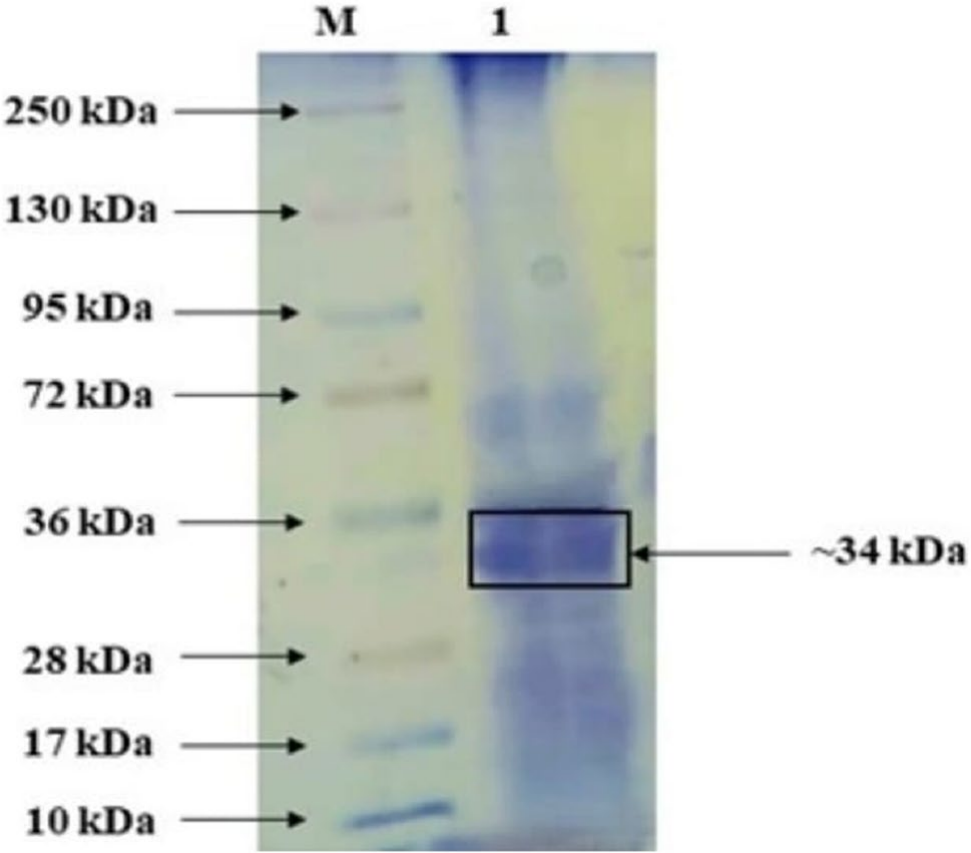
SDS-PAGE profile of the fraction containing putative bacteriocin-like protein (3B₃) obtained from *L. fermentum*. Lane M: protein molecular weight marker (10–250 kDa); Lane 1: FPLC fraction 3B₃ showing a prominent protein band at approximately 34 kDa, analyzed using GelAnalyzer 23.1.1 software

SDS-PAGE analysis was performed to estimate the molecular weight and assess the purity of the putative bacteriocin-like protein fraction (3B₃) obtained from *L. fermentum*. The gel profile shown in Fig. 11, analyzed using GelAnalyzer 23.1.1 software, showed a prominent band at approximately 34 kDa, indicating successful partial purification of the active compound.

## Discussion

In this study, 40 local LAB isolates were obtained from different sources, and their neutralized CFSs were tested to exclude the effect of organic acids. Only 22.5% of the isolates showed measurable anti-*H. pylori* activity. Among these, isolate N was the most effective, producing the largest mean inhibition zone (15.74 ± 0.26 mm) and exhibiting the lowest MIC and MBC values (93.7–357 µg/mL), suggesting strong bactericidal potential based on its recorded MBC/MIC ratio, which ranged from (1–2) across *H. pylori* groups. In contrast, the remaining isolates (L, 1PA, P, FA, DB, and DS) were less effective, showing the highest MIC and MBC values (375–750 µg/mL), while isolates M and C displayed an even higher MBC value 750 µg/mL, indicating weaker bactericidal effects. This variation in activity may be attributed to differences in the susceptibility of the tested *H. pylori* strains, which are known to display considerable genetic and phenotypic diversity influencing their resistance to antimicrobial agents.

To explore their potential in overcoming antibiotic resistance, the synergistic effects of the nine LAB-CFSs in combination with antibiotics were assessed. Four types of interactions were observed: synergism, antagonism, additive effects, and enhanced effects. Isolate N showed the highest overall activity, demonstrating synergistic effects with all antibiotics across all the tested *H. pylori* groups. In contrast, isolate DB displayed the weakest performance: it exhibited antagonistic interactions with clarithromycin, metronidazole, and amoxicillin against all groups, produced enhanced effects with tetracycline, rifampicin, and levofloxacin, and showed true synergism only with levofloxacin in group 2. These results indicate that the N extract is the most effective LAB-CFS for enhancing antibiotic activity against *H. pylori* across a broad spectrum of antibiotics. Based on these findings, the N isolate was subsequently selected for its identification and characterization. It was molecularly identified as *L. fermentum* through 16 S rRNA gene sequencing.

Many studies found to be in agreement with our finding in the concept of anti- *H. pylori* activity of *L. fermentum*, where García et al. (2017) [55], recorded that *L. fermentum* UCO-979 C has an inhibitory effect on the growth and urease activity of *H. pylori* strains. In addition, our results were more promising than those recorded by Sornsenee et al. (2024) [12], which reported that CFS of *L. fermentum* T0701 has anti-*H. pylori* activity with an inhibition zone diameter of only 10.67 mm while our strain recorded 15 ± 0.00: 16.66 ± 0.33 mm. Alongside the importance of the anti- *H. pylori* activity for our local isolate, it was an important issue to study its effect on the therapeutic antibiotics used at the *H. pylori* treatment protocol, To the best of our knowledge, published studies specifically investigating the synergistic activity of *L. fermentum* in combination with antibiotics against *H. pylori* are currently lacking. However, synergistic effects between antibiotics and other LAB species have been reported. However, synergistic effects have been demonstrated for other LAB species. For instance, Jin and Yang (2021) [56] demonstrated that *Lactobacillus salivarius* enhanced the efficacy of clarithromycin and amoxicillin against *H. pylori*. In addition, Ji and Yang (2021) [57] reported that *L. plantarum* LN66 could help in the eradication of *H. pylori* when added to therapy, including levofloxacin. These findings support the concept that LAB can modulate bacterial susceptibility to antibiotics. This study provides novel evidence that *L. fermentum* may elicit a synergistic effect similar to that of previously reported probiotic strains, thereby supporting its candidacy as a therapeutic adjunct in *H. pylori* management. Therefore, we assessed its potential probiotic criteria.

In parallel with molecular identification, the probiotic properties of the local LAB N isolate (*L. fermentum*) were evaluated using *L. plantarum* ATCC 8014 as a reference strain. Since probiotic efficacy largely depends on survival under gastrointestinal stress, including acidic conditions in the stomach and bile salts in the duodenum, which significantly affect LAB viability [58]. Acid and bile tolerance were assessed. Both *L. fermentum* and the reference strain demonstrated good survivability across acidic conditions (pH 1–4), with a similar trend observed in bile salt exposure (0.5–2%). In particular, both strains exhibited higher survival at pH 3 (55.77% for *L. fermentum* and 65.70% for *L. plantarum*) compared with pH 2 and pH 4, while the highest survival was recorded at pH 3 and 0.5% bile salts (78% and 76.17%, respectively), with maintained viability even at higher bile concentrations. The nonlinear pH response suggests that pH 3 may represent an optimal condition for activation of acid resistance mechanisms, whereas reduced stress signaling at pH 4 may account for the observed decrease in survival. Consistent with this pattern, the ability of both strains to maintain substantial survival under bile stress further supports their physiological adaptation to gastrointestinal conditions. These findings are in agreement with Prabhurajeshwar and Chandrakanth (2017) [59] and Falah et al. (2019) [60], who reported similar tolerance patterns in *L. fermentum*. Importantly, the acid tolerance of isolate N aligns with widely accepted probiotic selection criteria, where survival rates of approximately 50% or higher indicate acceptable acid resistance, and similarly, sustained survival above 50% under bile exposure further confirms its potential to withstand gastrointestinal transit and supports its probiotic candidacy.

Cell surface properties are critical for probiotic strains as they influence adhesion to gastrointestinal epithelial surfaces (Polak-Berecka et al., 2014) [61]. The adhesion potential of the isolates was assessed via surface hydrophobicity and auto-aggregation assays. *L. fermentum* exhibited slightly higher hydrophobicity than the reference strain in xylene (98.15 ± 0.57% vs. 97.38 ± 0.13%), while *L. plantarum* showed greater hydrophobicity in chloroform (98.15 ± 0.33% vs. 96.01 ± 0.19%). These findings indicate strong hydrophobic characteristics for both strains, supporting their potential to adhere to host epithelial surfaces. Our results align with previous studies reporting high hydrophobicity in *L. fermentum* strains with both solvents [62–63], While our results were in disagreement with **Li** et al., **(2015)** [64], **who** reported that *L. fermentum* GA4 showed the lowest hydrophobicity (0.86%) for xylene.

For auto-aggregation, our results showed that *L. fermentum* exhibited significantly higher activity (87.64 ± 0.82%) compared to the reference strain *L. plantarum* ATCC 8014 (63.45 ± 0.66%), suggesting that this property may enhance its ability to survive in sufficient numbers and effectively colonize the gastrointestinal tract. These values are substantially higher than those reported by Ramos et al. (2013) [65], who recorded 55.61% auto-aggregation for *L. fermentum* CH58, and by Rashad Hameed and Abdul Sattar Salman (2023) [66], who reported auto-aggregation rates of 57.14% and 14.89% for different *L. fermentum* strains.

Regarding co-aggregation, the ability of probiotic strains to co-aggregate with pathogenic bacteria is an important trait that may facilitate pathogen exclusion and colonization resistance in the gastrointestinal tract. In this study, *L. fermentum* demonstrated notable co-aggregation with *S. aureus* (61.69 ± 1.39%), *E. coli* (38.80 ± 0.58%), and *K. pneumoniae* (50.53 ± 1.37%). These values were comparable or superior to those of the reference strain *L. plantarum* ATCC 8014, which exhibited co-aggregation of 61.69 ± 0.80%, 39.79 ± 2.23%, and 37.82 ± 2.42%, respectively. Although co-aggregation with *H. pylori* was not tested, the ability of *L. fermentum* to interact with both Gram-positive and Gram-negative pathogens suggests a broad-spectrum co-aggregative potential. The higher co-aggregation with *K. pneumoniae* compared to the reference strain may reflect strain-specific surface properties such as variations in cell surface proteins or exopolysaccharides, which are known to influence microbial adhesion and aggregation. These features could play a critical role in supporting the competitive exclusion of pathogens in mucosal environments [67]. Our results are more promising than those reported by Alizadeh Behbahani et al. (2024) [68], who found 20.11 ± 0.47% co-aggregation of *L. fermentum* IMAU70160 with *S. aureus*, and by Berkes et al. (2020) [69], who reported 30% and 40% co-aggregation of *L. fermentum* Qi6 with *E. coli* and MRSA, respectively.

For the safety evaluation of *L. fermentum*, hemolytic activity was assessed according to the European Food Safety Authority (EFSA) guidelines, *L. fermentum* exhibited no hemolysis, consistent with previous reports [70–71]. In addition, antibiotic sensitivity, a critical factor for probiotic safety, was also evaluated. One potential concern is that antibiotic resistance genes could be horizontally transferred to pathogenic bacteria [72]. However, **Darsanaki** et al. **(2013)** [73] reported that probiotic strains carrying intrinsic resistance genes are not inherently hazardous, as these genes are typically non-mobilizable. In this regard, *L. fermentum*, was tested for its susceptibility against eight commonly used antibiotics. The isolate was sensitive to tetracycline, clindamycin, azithromycin, amoxicillin, and rifampicin, showed moderate sensitivity to levofloxacin, and was resistant to metronidazole and cephradine. Notably, *L. fermentum* exhibited high sensitivity to several antibiotics (e.g., amoxicillin, rifampicin, tetracycline), in contrast to *H. pylori*, which displayed multidrug resistance. Its resistance to metronidazole is consistent with previous study **(Kaewnopparat** et al., **2013)** [74] and is likely intrinsic and non-transferable. Therefore, it is assumed that co-administration of *L. fermentum* with metronidazole-containing regimens may allow the probiotic to survive and support gastrointestinal microbiota during therapy. Conversely, its moderate sensitivity to levofloxacin may limit its use alongside fluoroquinolone-based treatments unless strain survival is confirmed under treatment conditions; Alternatively, the purified bacteriocin from *L. fermentum* could be developed as a pharmaceutical agent and co-administered with existing therapies, provided that its safety toward normal host cells is verified, as indicated in previous study [75].

As this study targets *H. pylori* and its consequences, the cytotoxic activity of the protein precipitate containing a putative bacteriocin from *L. fermentum* was evaluated against Caco-2 using the MTT assay. Results demonstrated a concentration-dependent reduction in cell viability, with higher concentrations producing stronger cytotoxic effects. The IC₅₀ value was 19.73 ± 0.16 µg/mL, indicating potent activity against Caco-2 cells. Caco-2 cells were selected as a model due to their relevance in studying human colorectal adenocarcinoma and the potential link between *H. pylori* infection and colorectal cancer. Emerging evidence suggests that *H. pylori* may contribute to colorectal cancer progression by altering the colonic microbiome and modulating host immunity in both mouse models and human patients, with bacterial species associated with colorectal cancer being enriched upon infection [76]. Evaluating the cytotoxic effect of the protein precipitate containing a putative bacteriocin on Caco-2 cells provides preliminary evidence of its biological activity beyond antimicrobial effects and supports further investigation of its potential relevance to *H. pylori*-associated colorectal carcinogenesis.

The antibacterial activity of *L. fermentum* was primarily attributed to putative bacteriocin-like compounds present in the cell-free supernatant. which was detected using FPLC with monitoring at 220 and 280 nm. The chromatography yielded 22 fractions, three of which exhibited anti-*H. pylori* activity in subsequent bioassays, suggesting that antimicrobial peptide (bacteriocin-like) compounds may contribute to the observed activity. These findings align with studies by Sornsenee et al. (2024) [13], who observed strong anti-*H. pylori* activity in lyophilized cell-free supernatants containing bacteriocins, and Techo et al. (2019) [77], who demonstrated that *L. fermentum* P43-01 exhibits bacteriocin-mediated antimicrobial activity against *H. pylori* strains MS83 and BK364. In addition, SDS-PAGE analysis revealed a band at approximately 34 kDa, suggesting the presence of a higher molecular weight bacteriocin-like compound. This finding is consistent with a previous report by Wannun et al. (2016) [78], who demonstrated that *L. fermentum* SD11 produces Fermencin SD11 with a similar molecular weight (~ 34 kDa).

Overall, these findings demonstrate that CFS of *L. fermentum* strain M98, including a putative bacteriocin, exhibits potent inhibitory activity against clinical *H. pylori* isolates and also shows cytotoxic effects on Caco-2 cells. Collectively, these bioactivities suggest that this isolate may represent a promising multifunctional candidate with antimicrobial and potential anticancer-related applications. In addition, the observed synergistic interactions with multiple antibiotics further highlight its potential to enhance current eradication regimens and contribute to overcoming antimicrobial resistance, thereby supporting its relevance as a prospective adjunct therapeutic agent. Although the in vitro results obtained from human-derived *H. pylori* isolates are promising, further in vivo investigations and well-designed clinical trials are essential to evaluate its safety profile, biological stability, and therapeutic efficacy prior to clinical application.

## Conclusion

This study demonstrates that the cell-free supernatant (CFS) of *L. fermentum* strain M98, containing bacteriocin-like compounds, exhibits significant in vitro inhibitory activity against *H. pylori*, with variable efficacy among clinical isolates. Importantly, the CFS showed synergistic interactions when combined with selected conventional antibiotics, enhancing their antibacterial effectiveness. In addition, protein precipitate containing a putative bacteriocin from *L. fermentum* demonstrated cytotoxic activity against Caco-2 cells in vitro. Collectively, these findings highlight the potential of *L. fermentum* M98 as a promising adjunctive therapeutic or preventive agent against *H. pylori*, particularly in the context of increasing antibiotic resistance. Further in vivo studies are warranted to confirm its safety, efficacy, and therapeutic applicability.

## Supplementary Information

Supplementary Material 1.

## Data Availability

16 S rRNA gene sequence of Limosilactobacillus fermentum strain M98 was submitted to GenBank under the accession number PP940096.

## Abbreviations

APS: Ammonium persulfate
AX: Amoxicillin
AZM: Azithromycin
BLAST: Basic Local Alignment Search Tool
Caco-2: Colorectal adenocarcinoma cell line
CE: Cephradine
CFS: Cell-free supernatant
CLR: Clarithromycin
DA: Clindamycin
DMSO: Dimethyl Sulfoxide
FPLC: Fast protein liquid chromatography
IARC: International Agency for Research on Cancer
iTOL: Interactive Tree Of Life
LAB: Lactic acid bacteria
LEV: Levofloxacin
MALT lymphoma: Mucosa-associated lymphoid tissue lymphoma
MBC: Minimum bactericidal concentration
MET: Metronidazole
MHA: Müller-Hinton agar
MHB: Mueller–Hinton broth
MIC: Minimum inhibitory concentration
MRS: de Man, Rogosa, and Sharpe
MTT: 3-(4,5-dimethylthiazol-2-yl)-2,5-diphenyltetrazolium bromide
PBS: Phosphate buffer saline
RA: Rifampicin
RPMI: Roswell Park Memorial Institute
RUT: Rapid urease test
SDS-PAGE: Sodium Dodecyl Sulfate–Polyacrylamide Gel Electrophoresis
SE: Standard error
TE: Tetracycline
TEMED: Tetra-methyl-ethylene-diamine
