# Supplementary material for: Synergistic anti-Helicobacter pylori efficacy of a molecularly identified Limosilactobacillus fermentum isolate in combination with multiple antibiotics
Source: BMC Microbiol. 2026 Jul 4;26:591. doi: 10.1186/s12866-026-05350-8 (PMC13332602; doi:10.1186/s12866-026-05350-8)
Supplement: Supplementary file 1 — Supplementary Material 1. [file 12866_2026_5350_MOESM1_ESM.docx]

**Fig. S1.** Histogram represents the antibiotic sensitivity of the tested *H. pylori* isolates against its therapeutic antibiotics; MET (5μg): metronidazole; AX (25μg): amoxicillin; CLR (15μg): clarithromycin; TE (30μg): tetracycline; RA (30μg): rifampicin; LEV (5μg): levofloxacin.


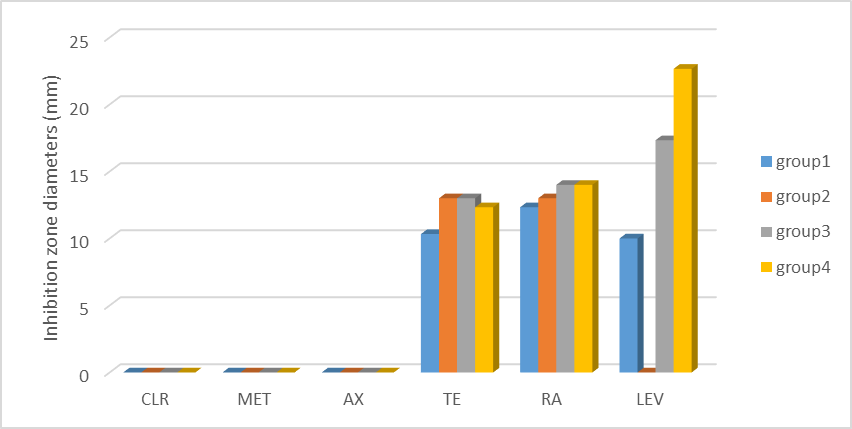


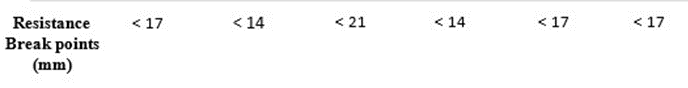


**Table S1.** The mean inhibition zone diameters (mm ± SE) of combined and separated inhibitory effects of DB-CFS with different antibiotics against *H. pylori* groups.

| ***H. pylori* group** | | **DB+CLR** | **DB+MET** | **DB+AX** | **DB+TE** | **DB+RA** | **DB+LEV** |
| --- | --- | --- | --- | --- | --- | --- | --- |
| 1 | **Combined action (mm)** | **14.66±0.33** | **13.33±0.33** | **14.66±0.33** | **20±0.00** | **16.66±0.33** | **23.66±0.33** |
|  | **Separated action (mm)** | ***DB=15.33 *CLR=0** | ***DB=15.33 *MET=0** | ***DB=15.33 *AX=0** | ***DB=15.33 *TE=10.33** | ***DB=15.33 *RA=12.33** | *** DB=15.33 *LEV=10** |
| 2 | **Combined action (mm)** | **12.66±0.33** | **0.00±0.00** | **0.00±0.00** | **20±0.00** | **16.66±0.34** | **30±0.00** |
|  | **Separated action (mm)** | ***DB=15.66 *MET=0** | ***DB=15.66 *MET=0** | ***DB=15.66* *AX=0** | ***DB=15.66 *TE=13** | *** DB=15.66 *RA=13** | *** DB=15.66 *LEV=0** |
| 3 | **Combined action (mm)** | **13.66±0.33** | **0.00±0.00** | **0.00±0.00** | **19.33±0.33** | **18±0.00** | **30±0.00** |
|  | **Separated action (mm)** | *** DB=16 *CLR=0** | *** DB=16 *MET=0** | *** DB=16 *AX=0** | *** DB=16 *TE=12.33** | *** DB=16 *RA=14** | *** DB=16 *LEV=17.33** |
| 4 | **Combined action (mm)** | **14±0.00** | **12±0.00** | **12±0.00** | **17±0.00** | **15±0.00** | **23±0.00** |
|  | **Separated action (mm)** | ***DB=15.66 *CLR=0** | ***DB=15.66 *MET=0** | ***DB=15.66 *AX=0** | ***DB=15.66 *TE=13** | *** DB=15.66 *RA=14** | *** DB=15.66 *LEV=22.66** |

**Table S2.** The mean inhibition zone diameters (mm ± SE) of combined and separated inhibitory effects of C-CFS with different antibiotics against *H. pylori* groups.

| ***H. pylori* group** | | **C+CLR** | **C+MET** | **C+AX** | **C+TE** | **C+RA** | **C+LEV** |
| --- | --- | --- | --- | --- | --- | --- | --- |
| 1 | **Combined action (mm)** | **11±0.00** | **11±0.00** | **10±0.00** | **27.66±0.33** | **18.33±0.33** | **32.33±0.33** |
|  | **Separated action (mm)** | ***C=15.66 *MET=0** | ***C=15.66 *MET=0** | ***C=15.66 *AX=0** | ***C=15.66 *TE=10.33** | *** C=15.66** ***RA=12.33** | ***C=15.66 *LEV=10** |
| 2 | **Combined action (mm)** | **0.00±0.00** | **0.00±0.00** | **0.00±0.00** | **20±0.00** | **17±0.00** | **30±0.00** |
|  | **Separated action (mm)** | *** C=16.33 *CLR=0** | ***C=16.33** ***MET=0** | ***C=16.33 *AX=0** | ***C=16.33 *TE=13** | *** C=16.33 *RA=13** | *** C=16.33 *LEV=0** |
| 3 | **Combined action (mm)** | **15±0.00** | **11.66±0.33** | **11.66±0.33** | **16.33±0.33** | **18±0.00** | **24±0.00** |
|  | **Separated action (mm)** | *** C=15.66 *CLR=0** | ***C=15.66 *MET=0** | ***C=15.66 *AX=0** | ***C=15.66 *TE=13** | *** C=15.66 *RA=14** | *** C=15.66 *LEV=17.33** |
| 4 | **Combined action (mm)** | **14.33±0.33** | **0.00±0.00** | **0.00±0.00** | **20±0.01** | **16±0.00** | **32±1** |
|  | **Separated action (mm)** | *** C=14.33 *CLR=0** | ***C=14.33 *MET=0** | ***C=14.33 *AX=0** | ***C=14.33 *TE=12.33** | *** C=14.33 *RA=14** | *** C=14.33 *LEV=22.66** |

**Table S3.** The mean inhibition zone diameters (mm ± SE) of combined and separated inhibitory effects of DS-CFS with different antibiotics against *H. pylori* groups.

| ***H. pylori* group** | | **DS+CLR** | **DS+MET** | **DS+AX** | **DS+TE** | **DS+RA** | **DS+LEV** |
| --- | --- | --- | --- | --- | --- | --- | --- |
| 1 | **Combined action (mm)** | **10±0.00** | **0.00±0.00** | **0.00±0.00** | **16±0.00** | **14.33±0.33** | **19.66±0.33** |
|  | **Separated action (mm)** | *** DS=13 *CLR=0** | *** DS=13 *MET=0** | *** DS=13 *AX=0** | *** DS=13 *TE=10.33** | *** DS=13 *RA=12.33** | *** DS=13 *LEV=10** |
| 2 | **Combined action (mm)** | **13±0.00** | **14.66±0.33** | **13.66±0.66** | **27.33±0.33** | **18±0.00** | **30±0.00** |
|  | **Separated action (mm)** | *** DS=16.66 *CLR=0** | ***DS=16.66 *MET=0** | ***DS=16.66 *AX=0** | ***DS=16.66 *TE=13** | *** DS=16.66 *RA=13** | ***DS=16.66 *LEV=0** |
| 3 | **Combined action (mm)** | **10.33±0.33** | **0.00±0.00** | **0.00±0.00** | **16±0.00** | **17.33±0.33** | **25±0.00** |
|  | **Separated action (mm)** | *** DS=19 *CLR=0** | *** DS=19 *MET=0** | *** DS=19 *AX=0** | *** DS=19 *TE=12.33** | *** DS=19 *RA=14** | *** DS=19 *LEV=17.33** |
| 4 | **Combined action (mm)** | **15.66±0.33** | **0.00±0.00** | **15.66±0.33** | **18±0.00** | **18±0.00** | **30±0.00** |
|  | **Separated action (mm)** | *** DS=13 *CLR=0** | *** DS=13 *MET=0** | *** DS=13 *AX=0** | *** DS=13 *TE=13** | *** DS=13 *RA=14** | *** DS=13 *LEV=22.66** |

**Table S4.** The mean inhibition zone diameters (mm ± SE) of combined and separated inhibitory effects of FA-CFS with different antibiotics against *H. pylori* groups.

| ***H. pylori* group** | | **FA+CLR** | **FA+MET** | **FA+AX** | **FA+TE** | **FA+RA** | **FA+LEV** |
| --- | --- | --- | --- | --- | --- | --- | --- |
| 1 | **Combined action (mm)** | **0.00±0.00** | **0.00±0.00** | **0.00±0.00** | **17.66±0.33** | **16±0.00** | **28±0.00** |
|  | **Separated action (mm)** | ***FA=15.33**  *** CLR=0** | ***FA=15.33 *MET=0** | ***FA=15.33 *AX=0** | ***FA=15.33 *TE=10.33** | ***FA=15.33 *RA=12.33** | *** FA=15.33**  *** LEV=10** |
| 2 | **Combined action (mm)** | **0.00±0.00** | **0.00±0.00** | **0.00±0.00** | **20±0.00** | **18±0.00** | **30±0.00** |
|  | **Separated action (mm)** | *** FA=14 *CLR=0** | *** FA=14 *MET=0** | *** FA=14 *AX=0** | *** FA=14 *TE=13** | *** FA=14 *RA=13** | *** FA=14 *LEV=0** |
| 3 | **Combined action (mm)** | **14.66±0.33** | **14.66±0.33** | **14.66±0.33** | **17±0.00** | **17.33±0.33** | **30±0.00** |
|  | **Separated action (mm)** | *** FA=13 *CLR=0** | *** FA=13 *MET=0** | *** FA=13 *AX=0** | *** FA=13 *TE=12.33** | *** FA=13 *RA=14** | *** FA=13 *LEV=17.33** |
| 4 | **Combined action (mm)** | **0.00±0.00** | **0.00±0.00** | **0.00±0.00** | **16±0.00** | **16±0.00** | **25.66±0.33** |
|  | **Separated action (mm)** | *** FA=17 *CLR=0** | *** FA=17 *MET=0** | *** FA=17 *AX=0** | *** FA=17 *TE=13** | ***FA=17 *RA=14** | * **FA=17 *LEV=22.66** |

**Table S5.** The mean inhibition zone diameters (mm ± SE) of combined and separated inhibitory effects of M-CFS with different antibiotics against *H. pylori* groups.

| ***H. pylori* group** | | **M+CLR** | **M+MET** | **M+AX** | **M+TE** | **M+RA** | **M+LEV** |
| --- | --- | --- | --- | --- | --- | --- | --- |
| 1 | **Combined action (mm)** | **0.00±0.00** | **0.00±0.00** | **0.00±0.00** | **25±0.00** | **15±0.00** | **29.66±0.33** |
|  | **Separated action (mm)** | *** M=13 *CLR=0** | *** M=13 *MET=0** | *** M=13 *AX=0** | *** M=13 *TE=10.33** | *** M=13** ***RA=12.33** | *** M=13 *LEV=10** |
| 2 | **Combined action (mm)** | **14±0.00** | **10±0.00** | **16.33±0.33** | **22.66±0.33** | **16.66±0.33** | **33.66±0.33** |
|  | **Separated action (mm)** | *** M=15.66 *CLR=0** | ***M=15.66 *MET=0** | ***M=15.66 *AX=0** | ***M=15.66 *TE=13** | ***M=15.66 *RA=13** | *** M=15.66 * LEV=0** |
| 3 | **Combined action (mm)** | **14.33±0.33** | **0.00±0.00** | **13.66±0.33** | **20±0.00** | **22±0.00** | **22.66±0.33** |
|  | **Separated action (mm)** | *** M=16.66 *CLR=0** | ***M=16.66 *MET=0** | ***M=16.66 *AX=0** | ***M=16.66 *TE=13** | ***M=16.66 *RA=14** | *** M=16.66 *LEV=17.33** |
| 4 | **Combined action (mm)** | **0.00±0.00** | **0.00±0.00** | **0.00±0.00** | **25±0.00** | **17±0.00** | **30±0.00** |
|  | **Separated action (mm)** | *** M=14 *CLR=0** | *** M=14 *MET=0** | *** M=14 *AX=0** | *** M=14 *TE=12.33** | *** M=14 *RA=14** | *** M=14 *LEV=22.66** |

**Table S6.** The mean inhibition zone diameters (mm ± SE) of combined and separated inhibitory effects of 1PA-CFS with different antibiotics against *H. pylori* groups.

| ***H. pylori* group** | | **1PA+CLR** | **1PA+MET** | **1PA+AX** | **1PA+TE** | **1PA+RA** | **1PA+LEV** |
| --- | --- | --- | --- | --- | --- | --- | --- |
| 1 | **Combined action (mm)** | **13.33±0.33** | **14.66±0.33** | **13.33±0.33** | **18.66±0.33** | **15.66±0.33** | **13±0.00** |
|  | **Separated action (mm)** | ***1PA=13 *CLR=0** | ***1PA=13 *MET=0** | ***1PA=13 *AX=0** | * **1PA=13** ***TE=10.33** | ***1PA=13 *RA=12.33** | *** 1PA=13 *LEV=10** |
| 2 | **Combined action (mm)** | **0.00±0.00** | **0.00±0.00** | **0.00±0.00** | **25±0.00** | **20±0.00** | **29.33±0.66** |
|  | **Separated action (mm)** | ***1PA=15.33 *CLR=0** | ***1PA=15.33 *MET=0** | ***1PA=15.33 *AX=0** | ***1PA=15.33 *TE=13** | ***1PA=15.33** ***RA=13** | *** 1PA=15.33 *LEV=0** |
| 3 | **Combined action (mm)** | **10±0.00** | **14.66±0.33** | **0.00±0.00** | **16.33±0.33** | **16.66±0.33** | **22.66±0.33** |
|  | **Separated action (mm)** | ***1PA=16.66 *CLR=0** | *** 1PA=16.66 * MET=0** | ***1PA=16.66 * AX=0** | ***1PA=16.66** * **TE=13** | ***1PA=16.66 *RA=14** | ***1PA=16.66 *LEV=17.33** |
| 4 | **Combined action (mm)** | **13±0.00** | **14.66±0.33** | **12±0.00** | **26±0.00** | **16±0.00** | **32±1** |
|  | **Separated action (mm)** | *** 1PA= 13.33 *CLR=0** | *** 1PA= 13.33 * MET=0** | *** 1PA= 13.33 * AX=0** | *** 1PA= 13.33 *TE=12.33** | *** 1PA= 13.33 *RA=14** | *** 1PA= 13.33 *LEV=22.66** |

**Table S7.** The mean inhibition zone diameters (mm ± SE) of combined and separated inhibitory effects of P-CFS with different antibiotics against *H. pylori* groups.

| ***H. pylori* group** | | **P+CLR** | **P+MET** | **P+AX** | **P+TE** | **P+RA** | **P+LEV** |
| --- | --- | --- | --- | --- | --- | --- | --- |
| 1 | **Combined action (mm)** | **15.66±0.33** | **15±0.00** | **15±0.00** | **27.66±0.66** | **18±0.00** | **29.33±0.66** |
|  | **Separated action (mm)** | *** P=16 *CLR=0** | *** P=16 *MET=0** | *** P=16 *AX=0** | *** P=16 *TE=10.33** | *** P=16 *RA=12.33** | *** P=16 *LEV=10** |
| 2 | **Combined action (mm)** | **13±0.00** | **12±0.00** | **12±0.00** | **25.66±0.33** | **15.66±0.33** | **30.33±0.33** |
|  | **Separated action (mm)** | *** P=14.66 *CLR=0** | ***P=14.66 *MET=0** | ***P=14.66 *AX=0** | ***P=14.66 *TE=13** | ***P=14.66 *RA=13** | ***P=14.66 *LEV=0** |
| 3 | **Combined action (mm)** | **12±0.00** | **10±0.00** | **0.00±0.00** | **16.33±0.33** | **29.66±0.00** | **30±0.00** |
|  | **Separated action (mm)** | ***P=15.33 *CLR=0** | *** P=15.33 *MET=0** | ***P=15.33** ***AX=0** | *** P=15.33 *TE=13** | ***P=15.33 *RA=14** | *** P=15.33 *LEV=17.33** |
| 4 | **Combined action (mm)** | **0.00±0.00** | **16.66±0.33** | **10.33±0.33** | **28.66±0.33** | **17±0.00** | **30±0.00** |
|  | **Separated action (mm)** | ***P=15.66 *CLR=0** | *** P=15.66 *MET=0** | ***P=15.66 *AX=0** | *** P=15.66 *TE=12.33** | ***P=15.66 *RA=14** | *** P=15.66 *LEV=22.66** |

**Table S8.** The mean inhibition zone diameters (mm ± SE) of combined and separated inhibitory effects of L-CFS with different antibiotics against *H. pylori* groups.

| ***H. pylori* group** | | **L+CLR** | **L+MET** | **L+AX** | **L+TE** | **L+RA** | **L+LEV** |
| --- | --- | --- | --- | --- | --- | --- | --- |
| 1 | **Combined action (mm)** | **15±0.00** | **10.66±0.33** | **13±0.00** | **19.66±0.33** | **17.66±0.33** | **30±0.00** |
|  | **Separated action (mm)** | * **L=10** ***CLR=0** | * **L=10** ***MET=0** | * **L=10** ***AX=0** | * **L=10** ***TE=10.33** | * **L=10 *RA=12.33** | * **L=10** ***LEV=10** |
| 2 | **Combined action (mm)** | **12±0.00** | **0.00±0.00** | **0.00±0.00** | **28.33±0.33** | **20±0.00** | **31.33±0.33** |
|  | **Separated action (mm)** | * **L=14.66 *CLR=0** | *** L=14.66** ***MET=0** | *** L=14.66** ***AX=0** | ***L=14.66 *TE=13** | *** L=14.66 *RA=13** | *** L=14.66 *LEV=0** |
| 3 | **Combined action (mm)** | **11.66±0.33** | **0.00±0.00** | **11.33±0.66** | **17±0.00** | **17±0.00** | **29±1** |
|  | **Separated action (mm)** | *** L=15 *CLR=0** | *** L=15 *MET=0** | *** L=15 *AX=0** | *** L=15 *TE=13** | *** L=15 *RA=14** | *** L=15 *LEV=17.33** |
| 4 | **Combined action (mm)** | **10±0.00** | **0.00±0.00** | **0.00±0.00** | **25.33±0.33** | **24.66±0.33** | **33.66±0.33** |
|  | **Separated action (mm)** | *** L=10 *CLR=0** | *** L=10 *MET=0** | *** L=10 *AX=0** | *** L=10 *TE=12.33** | *** L=10 *RA=14** | *** L=10 *LEV=22.66** |

**Table S9.** The mean inhibition zone diameters (mm ± SE) of combined and separated inhibitory effects of N-CFS with different antibiotics against *H. pylori* groups as reported by Ali et al., (2025).

| ***H. pylori*  group** | | **N+CLR** | **N+MET** | **N+AX** | **N+TE** | **N+RA** | **N+LEV** |
| --- | --- | --- | --- | --- | --- | --- | --- |
| 1 | **Combined action (mm)** | **14.33±0.00** | **14.33±0.33** | **14.33±0.33** | **29.66±0.33** | **29.66±0.33** | **30.33±0.33** |
|  | **Separated action (mm)** | ***N=15.66, *CLR= 0** | ***N=15.66 *MET=0** | ***N=15.66 *AX=0** | ***N=15.66 *TE=10.33** | ***N=15.66 *RA=12.33** | ***N=15.66 *LEV=10** |
| 2 | **Combined action (mm)** | **15.66±0.33** | **0.00±0.00** | **15.33±0.33** | **30.33±0.33** | **18±0.00** | **30.33±0.33** |
|  | **Separated action (mm)** | ***N=15, *CLR=0** | ***N=15 *MET=0** | ***N=15 *AX=0** | ***N=15 *TE=13** | ***N=15 *RA=13** | ***N=15 *LEV=0** |
| 3 | **Combined action (mm)** | **15.33±0.33** | **15±0.00** | **14.33±0.00** | **20.66±0.66** | **17.33±0.00** | **29.33±0.66** |
|  | **Separated action (mm)** | ***N=16.66, *CLR=0** | ***N=16.66 *MET=0** | ***N=16.66 *AX=0** | ***N=16.66 *TE=12.33** | ***N=16.66 *RA=14** | ***N=16.66 *LEV=17.33** |
| 4 | **Combined action (mm)** | **10±0.00** | **18.66±0.33** | **15.33±0.00** | **30.33±0.33** | **14.66±0.33** | **30±0.00** |
|  | **Separated action (mm)** | ***N=15.66, *CLR=0** | ***N=15.66 *MET=0** | ***N=15.66 *AX=0** | ***N=15.66 *TE=13** | ***N=15.66 *RA=14** | ***N=15.66 *LEV=22.66** |

| **CFS** | **Antibiotics** | **p-value** |
| --- | --- | --- |
| N | All | 0.000 |
| L | All | 0.000 |
| P | All | 0.000 |
| 1PA | All | 0.000 |
| FA | All | 0.000 |
| M | All | 0.000 |
| C | All | 0.000 |
| DB | All | 0.000 |
| DS | All | 0.000 |

**Table S10**. P-values obtained from one-way ANOVA analysis of inhibition zone differences for combinations of LAB cell-free supernatants (CFSs) and antibiotics against four ***H. pylori*** groups. All values indicate statistical significance (p < 0.05).

**Table S11**. Represents the survival rates of N isolate and *L. plantarum ATCC8014* to different pH values and bile salt concentrations.

| **Tested**  **conc.**  **Isolates** | **Bile salt survival rate (%)** | | | | **pH survival rate (%)** | | | |
| --- | --- | --- | --- | --- | --- | --- | --- | --- |
|  | **0.5%** | **1%** | **1.5%** | **2%** | **1** | **2** | **3** | **4** |
| N isolate | **78** | 71.5 | 70.22 | 68.44 | 32.66 | 46.66 | **55.77** | 48.22 |
| *L. plantarum* ATCC 8014 | **76.17** | 64.65 | 58.9 | 51.3 | 39.26 | 47.9 | **65.70** | 57.32 |

**Fig. S2.** Represents the combination effect between DB extract with antibiotics against different *H. pylori* isolates groups. A represents group 1 of *H. pylori* isolates; B represents group 2 of *H. pylori* isolates; C represents group 3 of *H. pylori* isolates; D represents group 4 of *H. pylori* isolates. Each number from (1-6) represents the tested antibiotics,1= clarithromycin (CLR,15μg); 2= metronidazole (MET, 5μg); 3= amoxicillin (AX, 25μg); 4= tetracycline (TE, 30μg); 5= rifampicin (RA, 30μg); 6= levofloxacin (LEV, 5μg).


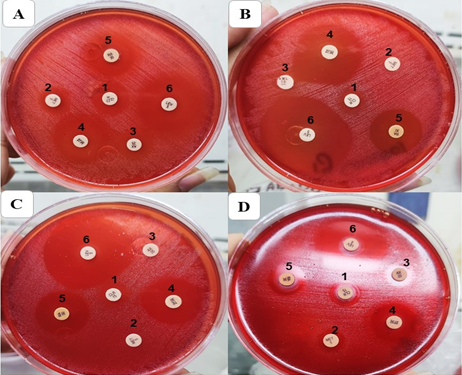


**Fig. S3.** Represents the combination effect between the C extract with antibiotics against different *H. pylori* isolates groups. A represents group 1 of *H. pylori* isolates; B represents group 2 of *H. pylori* isolates; C represents group 3 of *H. pylori* isolates; D represents group 4 of *H. pylori* isolates. Each number from (1-6) represents the tested antibiotics,1= clarithromycin (CLR,15μg); 2= metronidazole (MET, 5μg); 3= amoxicillin (AX, 25μg); 4= tetracycline (TE, 30μg); 5= rifampicin (RA, 30μg); 6= levofloxacin (LEV, 5μg).


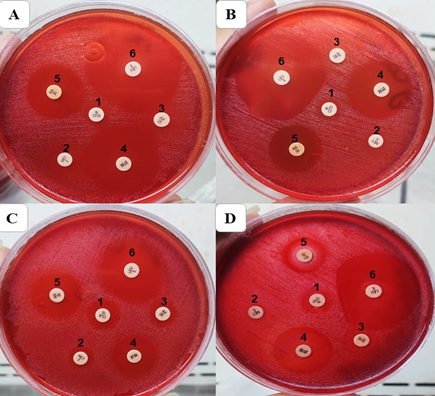


**Fig. S4.** Represents the combination effect between DS extract with antibiotics against different *H. pylori* isolates groups. A represents group 1 of *H. pylori* isolates; B represents group 2 of *H. pylori* isolates; C represents group 3 of *H. pylori* isolates; D represents group 4 of *H. pylori* isolates. Each number from (1-6) represents the tested antibiotics,1= clarithromycin (CLR,15μg); 2= metronidazole (MET, 5μg); 3= amoxicillin (AX, 25μg); 4= tetracycline (TE, 30μg); 5= rifampicin (RA, 30μg); 6= levofloxacin (LEV, 5μg).


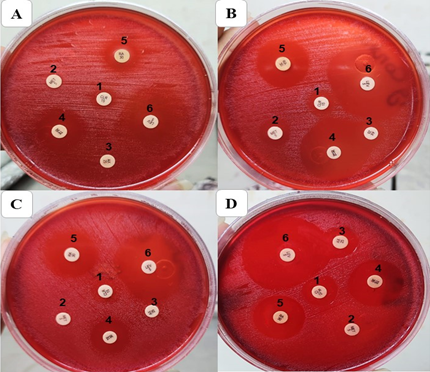


**Fig. S5.** Represents the combination effect between FA extract with antibiotics against different *H. pylori* isolates groups. A represents group 1 of *H. pylori* isolates; B represents group 2 of *H. pylori* isolates; C represents group 3 of *H. pylori* isolates; D represents group 4 of *H. pylori* isolates. Each number from (1-6) represents the tested antibiotics,1= clarithromycin (CLR,15μg); 2= metronidazole (MET, 5μg); 3= amoxicillin (AX, 25μg); 4= tetracycline (TE, 30μg); 5= rifampicin (RA, 30μg); 6= levofloxacin (LEV, 5μg).


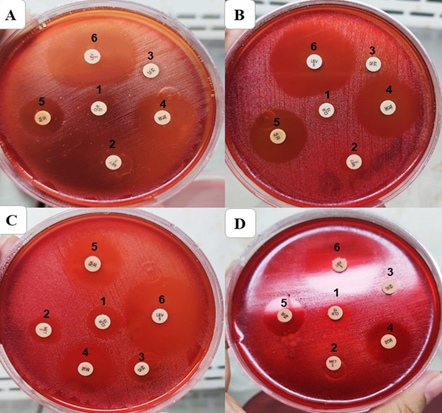


**Fig. S6.** Represents the combination effect between M extract with antibiotics against different *H. pylori* isolates groups. A represents group 1 of *H. pylori* isolates; B represents group 2 of *H. pylori* isolates; C represents group 3 of *H. pylori* isolates; D represents group 4 of *H. pylori* isolates. Each number from (1-6) represents the tested antibiotics,1= clarithromycin (CLR,15μg); 2= metronidazole (MET, 5μg); 3= amoxicillin (AX, 25μg); 4= tetracycline (TE, 30μg); 5= rifampicin (RA, 30μg); 6= levofloxacin (LEV, 5μg).


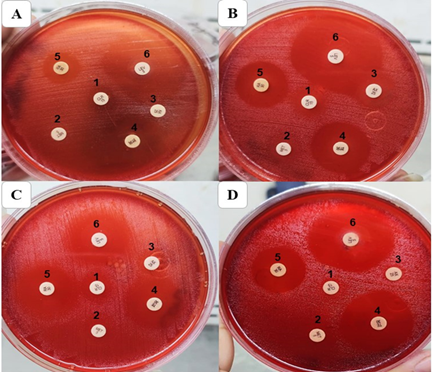


**Fig. S7.** Represents the combination effect between 1PA extract with antibiotics against different *H. pylori* isolates groups. A represents group 1 of *H. pylori* isolates; B represents group 2 of *H. pylori* isolates; C represents group 3 of *H. pylori* isolates; D represents group 4 of *H. pylori* isolates. Each number from (1-6) represents the tested antibiotics,1= clarithromycin (CLR,15μg); 2= metronidazole (MET, 5μg); 3= amoxicillin (AX, 25μg); 4= tetracycline (TE, 30μg); 5= rifampicin (RA, 30μg); 6= levofloxacin (LEV, 5μg).


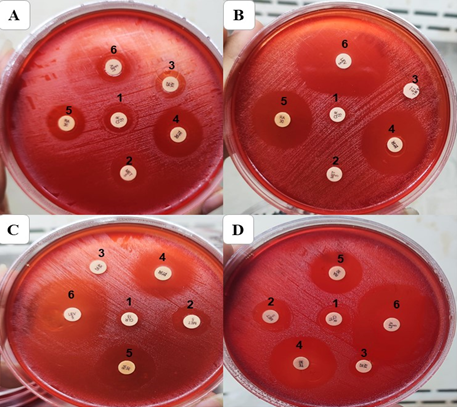


**Fig. S8.** Represents the combination effect between P extract with antibiotics against different *H. pylori* isolates groups. A represents group 1 of *H. pylori* isolates; B represents group 2 of *H. pylori* isolates; C represents group 3 of *H. pylori* isolates; D represents group 4 of *H. pylori* isolates. Each number from (1-6) represents the tested antibiotics,1= clarithromycin (CLR,15μg); 2= metronidazole (MET, 5μg); 3= amoxicillin (AX, 25μg); 4= tetracycline (TE, 30μg); 5= rifampicin (RA, 30μg); 6= levofloxacin (LEV, 5μg).


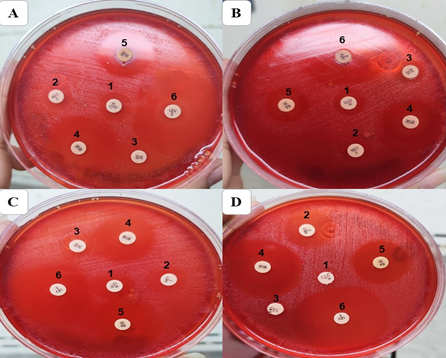


**Fig. S9.** Represents the combination effect between L extract with antibiotics against different *H. pylori* isolates groups. A represents group 1 of *H. pylori* isolates; B represents group 2 of *H. pylori* isolates; C represents group 3 of *H. pylori* isolates; D represents group 4 of *H. pylori* isolates. Each number from (1-6) represents the tested antibiotics,1= clarithromycin (CLR,15μg); 2= metronidazole (MET, 5μg); 3= amoxicillin (AX, 25μg); 4= tetracycline (TE, 30μg); 5= rifampicin (RA, 30μg); 6= levofloxacin (LEV, 5μg).


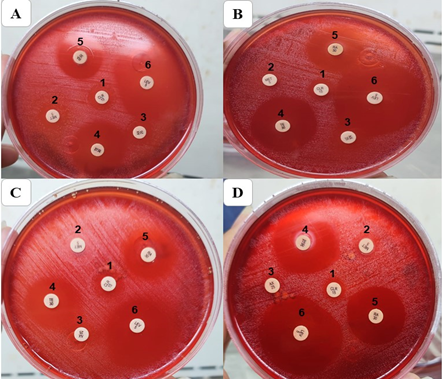


**Fig. S10.** Blood agar plate inoculated with both the isolate N and *L. plantarum* ATCC 8014 (a reference strain) exhibiting non-hemolytic behavior, with intact red blood cells around colonies.


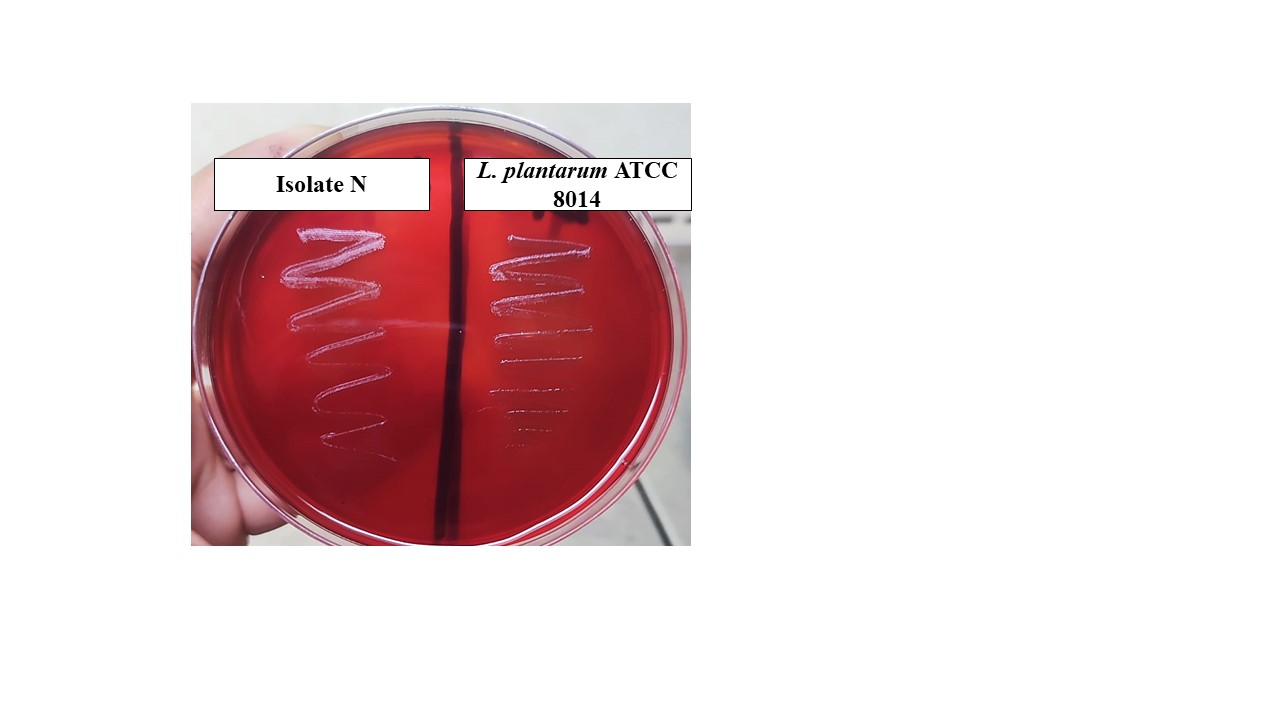


**Fig. S11.** Original full-length unprocessed image corresponding to Fig. 8A showing the disc diffusion assay of isolate N on Mueller–Hinton agar (MHA). Antibiotics tested: (1) Clindamycin, (2) Rifampicin, (3) Tetracycline, (4) Metronidazole, (5) Amoxicillin, (6) Azithromycin, (7) Levofloxacin, and (8) Cephradine.


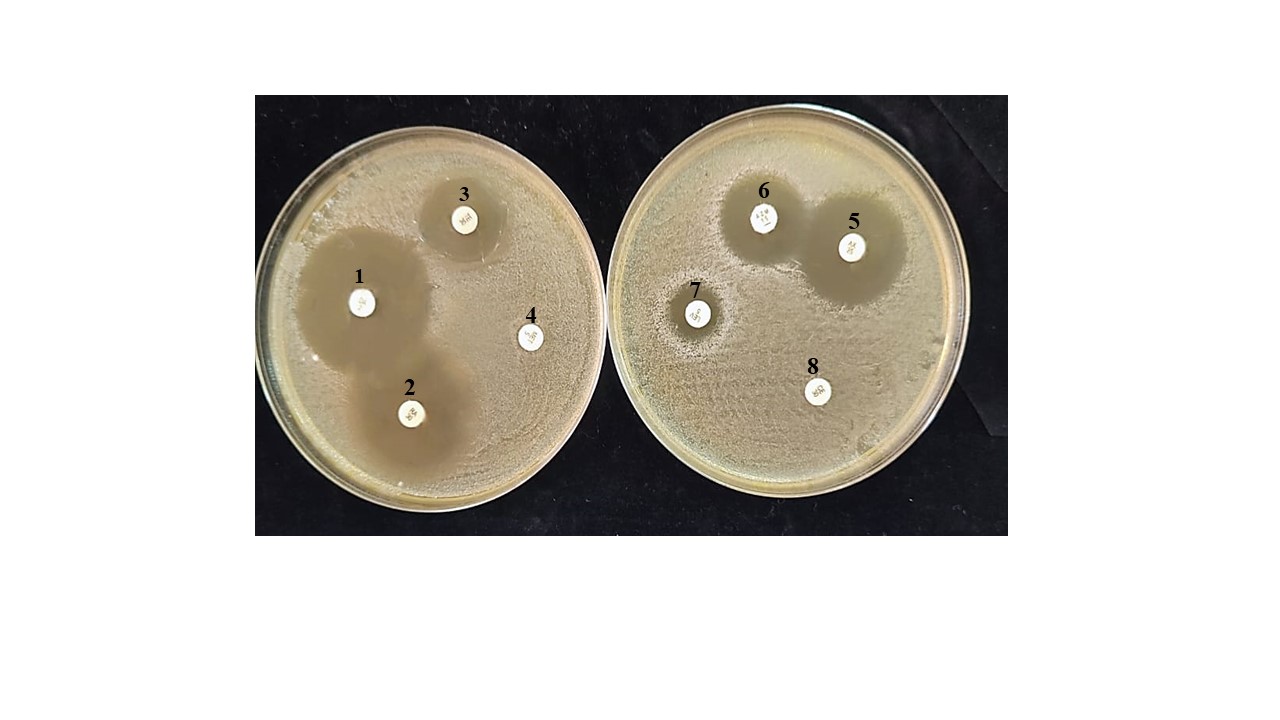


**Fig. S12**. Full-length original unprocessed agarose gel image corresponding to Fig. 9A in the manuscript. L: molecular weight marker (100 bp ladder); P: positive control; N: negative control; S: sample.


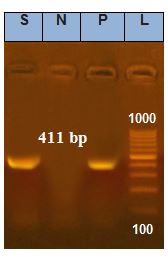


**Fig. S13.** Dose-dependent anticancer activity of isolate *L. fermentum* -derived protein precipitate, containing the bacteriocin, against the Caco-2 cell line treated with different concentrations. (A) Untreated control Caco-2 cell morphology. (B) Caco-2 cells treated with precipitated proteins (containing bacteriocin), exhibiting dose-dependent cytotoxic effects compared to the control.

**
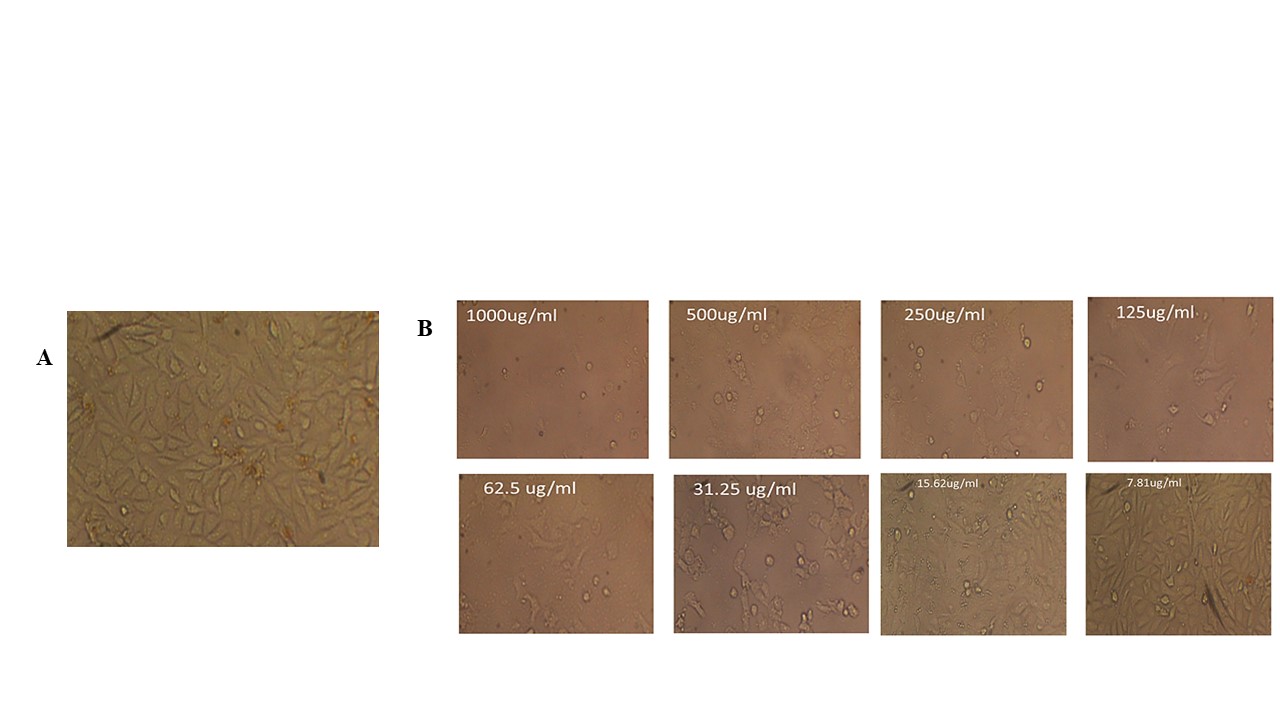
**

**Fig. S14**. Full-length original unprocessed FPLC chromatogram corresponding to Fig. 11A in the manuscript.


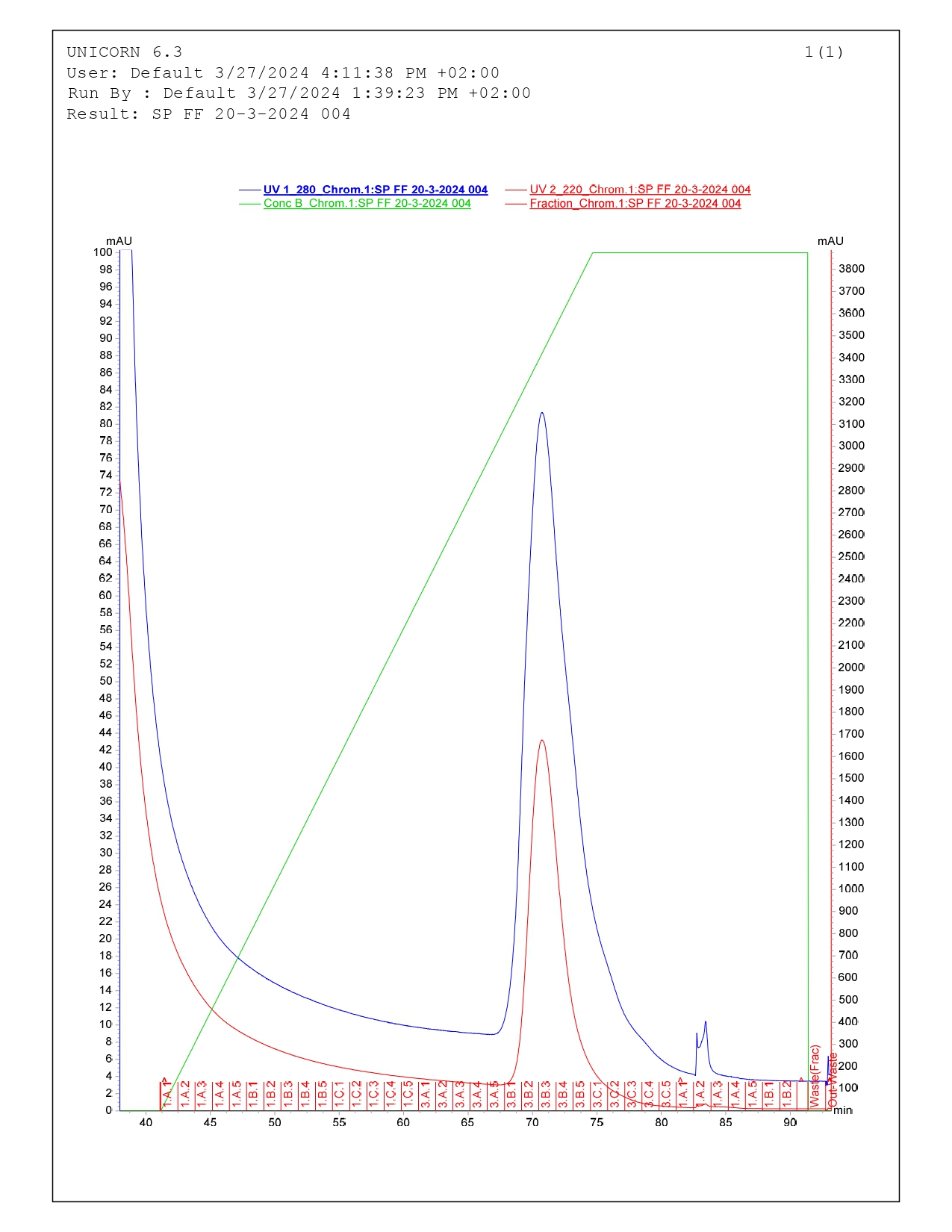


|  |  |
| --- | --- |
|  |  |
|  |  |
|  |  |
|  |  |
|  |  |
|  |  |
|  |  |
